# Supplementary material for: Quantifying the roles of vomiting, diarrhea, and residents vs. staff in norovirus transmission in U.S. nursing home outbreaks
Source: PLoS Comput Biol. 2020 Mar 25;16(3):e1007271. doi: 10.1371/journal.pcbi.1007271 (PMC7135310; doi:10.1371/journal.pcbi.1007271)
Supplement: S1 RMarkdown — This file contains instuctions and R code for estimating individual reproduction numbers. (HTML) [file pcbi.1007271.s002.html]

Estimating Individual Reproduction Numbers


# Estimating Individual Reproduction Numbers

#### *Carly Adams*

Load the required package, EpiEstim.

```
library(EpiEstim)
```

First, create numeric vectors for each outbreak. Each number in a vector represents the number of cases reporting illness onset on a given day of that outbreak. For example, for outbreak 1, 1 case reported illness onset on the first day of the outbreak, 0 cases reported illness onset on the second and third days of the outbreak, 4 cases reported illness onset on the fourth day of the outbreak, and so on.

```
outbreak1 <- c(1,0,0,4,8,2,3,5,0,0,3,1)
outbreak2 <- c(3,2,2,0,0,1,2,0,0,1)
outbreak3 <- c(1,0,0,8,0,1,1,0,0,22,5,4,4)
outbreak4 <- c(1,0,0,0,0,2,18,17,6,1,0,0,0,2,0,2,0,2)
outbreak5 <- c(2,0,4,6,7,8,1,1,1,0,0,2)
outbreak6 <- c(1,0,7,3,6,8,9,1,4,1,0,1)
```

Second, use the Wallinga Teunis (WT) method to estimate reproduction numbers, R, for illness onset dates of each outbreak.

The arguments “T.Start” and “T.End” are vectors of positive integers giving the starting and ending times of each window over which the reproduction numbers are estimated. This begins on day 1 for each outbreak and ends on the last day of the outbreak. For example, outbreak1 lasted 12 days, so T.Start and T.End both equal 1:12.

Using argument “method=ParametricSI” indicates that the serial interval (i.e., the time between symptom onset in primary cases and the secondary cases they generate) has a continuous distribution with a known mean and standard deviation. Indicate that the serial interval is gamma distributed with a mean of 3.6 days and a standard deviation of 2.0 days (this distribution was derived from several large norovirus outbreaks in child daycare centers in Sweden).

Use the “nSim” argument to set the number of simulated epidemic trees used for computation of the confidence intervals of the reproduction number to 10,000.

```
#Outbreak 1
WT1 <- WT(outbreak1, T.Start=1:12, T.End=1:12, method="ParametricSI", Mean.SI=3.6, Std.SI=1.998, plot=FALSE, nSim=10000)

#Outbreak 2
WT2 <- WT(outbreak2, T.Start=1:10, T.End=1:10, method="ParametricSI", Mean.SI=3.6, Std.SI=1.998, plot=FALSE, nSim=10000)
```

```
## Warning in WT(outbreak2, T.Start = 1:10, T.End = 1:10, method = "ParametricSI", : The serial interval distribution you have chosen is very wide compared to the duration of the epidemic.
## Estimation will be performed anyway but restults should be interpreted with care.
```

```
#Outbreak 3
WT3 <- WT(outbreak3, T.Start=1:13, T.End=1:13, method="ParametricSI", Mean.SI=3.6, Std.SI=1.998, plot=FALSE, nSim=10000)

#Outbreak 4
WT4 <- WT(outbreak4, T.Start=1:18, T.End=1:18, method="ParametricSI", Mean.SI=3.6, Std.SI=1.998, plot=FALSE, nSim=10000)

#Outbreak 5
WT5 <- WT(outbreak5, T.Start=1:12, T.End=1:12, method="ParametricSI", Mean.SI=3.6, Std.SI=1.998, plot=FALSE, nSim=10000)

#Outbreak 6
WT6 <- WT(outbreak6, T.Start=1:12, T.End=1:12, method="ParametricSI", Mean.SI=3.6, Std.SI=1.998, plot=FALSE, nSim=10000)
```

Third, bind the estimated reproduction numbers and corresponding lower and upper 95% confidence intervals together for each outbreak and save as a CSV file.

```
#Outbreak 1
outbreak1 <- cbind(WT1$R$`Mean(R)`,WT1$R$`Quantile.0.025(R)`,WT1$R$`Quantile.0.975(R)`,(WT1$R$`Std(R)`)^2)
write.csv(outbreak1, "outbreak1_R_estimates.csv")

#Outbreak 2
outbreak2 <- cbind(WT2$R$`Mean(R)`,WT2$R$`Quantile.0.025(R)`,WT2$R$`Quantile.0.975(R)`,(WT2$R$`Std(R)`)^2)
write.csv(outbreak2, "outbreak2_R_estimates.csv")

#Outbreak 3
outbreak3 <- cbind(WT3$R$`Mean(R)`,WT3$R$`Quantile.0.025(R)`,WT3$R$`Quantile.0.975(R)`,(WT3$R$`Std(R)`)^2)
write.csv(outbreak3, "outbreak3_R_estimates.csv")

#Outbreak 4
outbreak4 <- cbind(WT4$R$`Mean(R)`,WT4$R$`Quantile.0.025(R)`,WT4$R$`Quantile.0.975(R)`,(WT4$R$`Std(R)`)^2)
write.csv(outbreak4, "outbreak4_R_estimates.csv")

#Outbreak 5
outbreak5 <- cbind(WT5$R$`Mean(R)`,WT5$R$`Quantile.0.025(R)`,WT5$R$`Quantile.0.975(R)`,(WT5$R$`Std(R)`)^2)
write.csv(outbreak5, "outbreak5_R_estimates.csv")

#Outbreak 6
outbreak6 <- cbind(WT6$R$`Mean(R)`,WT6$R$`Quantile.0.025(R)`,WT6$R$`Quantile.0.975(R)`,(WT6$R$`Std(R)`)^2)
write.csv(outbreak6, "outbreak6_R_estimates.csv")
```

The last step is to assign reproduction numbers, 95% CIs, and variances to all cases involved in the 6 outbreaks. This has already been done for you in the provided file: “S1\_File.csv”. However, were you to do this yourself, you would open the CSV files created for each outbreak, which contain estimated reproduction numbers, corresponding 95% CIs, and variances (standard deviations squared) for each illness onset date, and also open the file with line lists combined from all outbreaks (“S1\_File.csv”). You would create four new columns in the line list titled “R”, “Lower CI”, and “Upper CI”, and “Variance” and manually copy and paste the estimated reproduction numbers, corresponding 95% CIs, and variances into the rows corresponding to the correct onset day. Cases with the same days of illness onset, within a given outbreak, would be assigned identical reproduction numbers, 95% CIs, and variances.

For example, in outbreak 1, the second day that cases were reported corresponds to the fourth day into the outbreak. On the fourth day into outbreak 1, four cases were reported. All four cases would be assigned the reproduction number and 95% CIs corresponding to the second day that cases were reported: R = 2.16 (95% CI: 1.25, 3) and variance = 0.24.
