# Supplementary material for: Quantifying the roles of vomiting, diarrhea, and residents vs. staff in norovirus transmission in U.S. nursing home outbreaks
Source: PLoS Comput Biol. 2020 Mar 25;16(3):e1007271. doi: 10.1371/journal.pcbi.1007271 (PMC7135310; doi:10.1371/journal.pcbi.1007271)
Supplement: S2 RMarkdown — This file contains instuctions and R code for estimating individual reproduction numbers using different serial interval lengths. (HTML) [file pcbi.1007271.s003.html]

Sensitivity Analysis: Estimating Individual Reproduction Numbers Using Different Serial Interval Lengths


# Sensitivity Analysis: Estimating Individual Reproduction Numbers Using Different Serial Interval Lengths

#### *Carly Adams*

Load the required package, EpiEstim.

```
library(EpiEstim)
```

First, create numeric vectors for each outbreak. Each number in a vector represents the number of cases reporting illness onset on a given day of that outbreak. For example, for outbreak 1, 1 case reported illness onset on the first day of the outbreak, 0 cases reported illness onset on the second and third days of the outbreak, 4 cases reported illness onset on the fourth day of the outbreak, and so on.

```
outbreak1 <- c(1,0,0,4,8,2,3,5,0,0,3,1)
outbreak2 <- c(3,2,2,0,0,1,2,0,0,1)
outbreak3 <- c(1,0,0,8,0,1,1,0,0,22,5,4,4)
outbreak4 <- c(1,0,0,0,0,2,18,17,6,1,0,0,0,2,0,2,0,2)
outbreak5 <- c(2,0,4,6,7,8,1,1,1,0,0,2)
outbreak6 <- c(1,0,7,3,6,8,9,1,4,1,0,1)
```

Second, use the Wallinga Teunis (WT) method to estimate reproduction numbers, R, for illness onset dates of each outbreak.

The arguments “T.Start” and “T.End” are vectors of positive integers giving the starting and ending times of each window over which the reproduction numbers are estimated. This begins on day 1 for each outbreak and ends on the last day of the outbreak. For example, outbreak1 lasted 12 days, so T.Start and T.End both equal 1:12.

Using argument “method=ParametricSI” indicates that the serial interval (i.e., the time between symptom onset in primary cases and the secondary cases they generate) has a continuous distribution with a known mean and standard deviation.

Use the “nSim” argument to set the number of simulated epidemic trees used for computation of the confidence intervals of the reproduction number to 10,000.

Indicate that the serial interval is gamma distributed, but change the mean from 3.6 days to the following serial interval lengths: 1.5 to 4 days by 0.5 day increments. Keep the standard deviation set to 2.0 days.

```
##################################### Mean Serial Interval of 1.5 Days ##################################### 
#Outbreak 1
WT1_1.5 <- WT(outbreak1, T.Start=1:12, T.End=1:12, method="ParametricSI", Mean.SI=1.5, Std.SI=1.998, plot=FALSE, nSim=10000)

#Outbreak 2
WT2_1.5 <- WT(outbreak2, T.Start=1:10, T.End=1:10, method="ParametricSI", Mean.SI=1.5, Std.SI=1.998, plot=FALSE, nSim=10000)
```

```
## Warning in WT(outbreak2, T.Start = 1:10, T.End = 1:10, method = "ParametricSI", : The serial interval distribution you have chosen is very wide compared to the duration of the epidemic.
## Estimation will be performed anyway but restults should be interpreted with care.
```

```
#Outbreak 3
WT3_1.5 <- WT(outbreak3, T.Start=1:13, T.End=1:13, method="ParametricSI", Mean.SI=1.5, Std.SI=1.998, plot=FALSE, nSim=10000)

#Outbreak 4
WT4_1.5 <- WT(outbreak4, T.Start=1:18, T.End=1:18, method="ParametricSI", Mean.SI=1.5, Std.SI=1.998, plot=FALSE, nSim=10000)

#Outbreak 5
WT5_1.5 <- WT(outbreak5, T.Start=1:12, T.End=1:12, method="ParametricSI", Mean.SI=1.5, Std.SI=1.998, plot=FALSE, nSim=10000)

#Outbreak 6
WT6_1.5 <- WT(outbreak6, T.Start=1:12, T.End=1:12, method="ParametricSI", Mean.SI=1.5, Std.SI=1.998, plot=FALSE, nSim=10000)


##################################### Mean Serial Interval of 2.0 Days ##################################### 
#Outbreak 1
WT1_2.0 <- WT(outbreak1, T.Start=1:12, T.End=1:12, method="ParametricSI", Mean.SI=2.0, Std.SI=1.998, plot=FALSE, nSim=10000)

#Outbreak 2
WT2_2.0 <- WT(outbreak2, T.Start=1:10, T.End=1:10, method="ParametricSI", Mean.SI=2.0, Std.SI=1.998, plot=FALSE, nSim=10000)
```

```
## Warning in WT(outbreak2, T.Start = 1:10, T.End = 1:10, method = "ParametricSI", : The serial interval distribution you have chosen is very wide compared to the duration of the epidemic.
## Estimation will be performed anyway but restults should be interpreted with care.
```

```
#Outbreak 3
WT3_2.0 <- WT(outbreak3, T.Start=1:13, T.End=1:13, method="ParametricSI", Mean.SI=2.0, Std.SI=1.998, plot=FALSE, nSim=10000)

#Outbreak 4
WT4_2.0 <- WT(outbreak4, T.Start=1:18, T.End=1:18, method="ParametricSI", Mean.SI=2.0, Std.SI=1.998, plot=FALSE, nSim=10000)

#Outbreak 5
WT5_2.0 <- WT(outbreak5, T.Start=1:12, T.End=1:12, method="ParametricSI", Mean.SI=2.0, Std.SI=1.998, plot=FALSE, nSim=10000)

#Outbreak 6
WT6_2.0 <- WT(outbreak6, T.Start=1:12, T.End=1:12, method="ParametricSI", Mean.SI=2.0, Std.SI=1.998, plot=FALSE, nSim=10000)


##################################### Mean Serial Interval of 2.5 Days ##################################### 
#Outbreak 1
WT1_2.5 <- WT(outbreak1, T.Start=1:12, T.End=1:12, method="ParametricSI", Mean.SI=2.5, Std.SI=1.998, plot=FALSE, nSim=10000)

#Outbreak 2
WT2_2.5 <- WT(outbreak2, T.Start=1:10, T.End=1:10, method="ParametricSI", Mean.SI=2.5, Std.SI=1.998, plot=FALSE, nSim=10000)
```

```
## Warning in WT(outbreak2, T.Start = 1:10, T.End = 1:10, method = "ParametricSI", : The serial interval distribution you have chosen is very wide compared to the duration of the epidemic.
## Estimation will be performed anyway but restults should be interpreted with care.
```

```
#Outbreak 3
WT3_2.5 <- WT(outbreak3, T.Start=1:13, T.End=1:13, method="ParametricSI", Mean.SI=2.5, Std.SI=1.998, plot=FALSE, nSim=10000)

#Outbreak 4
WT4_2.5 <- WT(outbreak4, T.Start=1:18, T.End=1:18, method="ParametricSI", Mean.SI=2.5, Std.SI=1.998, plot=FALSE, nSim=10000)

#Outbreak 5
WT5_2.5 <- WT(outbreak5, T.Start=1:12, T.End=1:12, method="ParametricSI", Mean.SI=2.5, Std.SI=1.998, plot=FALSE, nSim=10000)

#Outbreak 6
WT6_2.5 <- WT(outbreak6, T.Start=1:12, T.End=1:12, method="ParametricSI", Mean.SI=2.5, Std.SI=1.998, plot=FALSE, nSim=10000)


##################################### Mean Serial Interval of 3.0 Days ##################################### 
#Outbreak 1
WT1_3.0 <- WT(outbreak1, T.Start=1:12, T.End=1:12, method="ParametricSI", Mean.SI=3.0, Std.SI=1.998, plot=FALSE, nSim=10000)

#Outbreak 2
WT2_3.0 <- WT(outbreak2, T.Start=1:10, T.End=1:10, method="ParametricSI", Mean.SI=3.0, Std.SI=1.998, plot=FALSE, nSim=10000)
```

```
## Warning in WT(outbreak2, T.Start = 1:10, T.End = 1:10, method = "ParametricSI", : The serial interval distribution you have chosen is very wide compared to the duration of the epidemic.
## Estimation will be performed anyway but restults should be interpreted with care.
```

```
#Outbreak 3
WT3_3.0 <- WT(outbreak3, T.Start=1:13, T.End=1:13, method="ParametricSI", Mean.SI=3.0, Std.SI=1.998, plot=FALSE, nSim=10000)

#Outbreak 4
WT4_3.0 <- WT(outbreak4, T.Start=1:18, T.End=1:18, method="ParametricSI", Mean.SI=3.0, Std.SI=1.998, plot=FALSE, nSim=10000)

#Outbreak 5
WT5_3.0 <- WT(outbreak5, T.Start=1:12, T.End=1:12, method="ParametricSI", Mean.SI=3.0, Std.SI=1.998, plot=FALSE, nSim=10000)

#Outbreak 6
WT6_3.0 <- WT(outbreak6, T.Start=1:12, T.End=1:12, method="ParametricSI", Mean.SI=3.0, Std.SI=1.998, plot=FALSE, nSim=10000)


##################################### Mean Serial Interval of 3.5 Days ##################################### 
#Outbreak 1
WT1_3.5 <- WT(outbreak1, T.Start=1:12, T.End=1:12, method="ParametricSI", Mean.SI=3.5, Std.SI=1.998, plot=FALSE, nSim=10000)

#Outbreak 2
WT2_3.5 <- WT(outbreak2, T.Start=1:10, T.End=1:10, method="ParametricSI", Mean.SI=3.5, Std.SI=1.998, plot=FALSE, nSim=10000)
```

```
## Warning in WT(outbreak2, T.Start = 1:10, T.End = 1:10, method = "ParametricSI", : The serial interval distribution you have chosen is very wide compared to the duration of the epidemic.
## Estimation will be performed anyway but restults should be interpreted with care.
```

```
#Outbreak 3
WT3_3.5 <- WT(outbreak3, T.Start=1:13, T.End=1:13, method="ParametricSI", Mean.SI=3.5, Std.SI=1.998, plot=FALSE, nSim=10000)

#Outbreak 4
WT4_3.5 <- WT(outbreak4, T.Start=1:18, T.End=1:18, method="ParametricSI", Mean.SI=3.5, Std.SI=1.998, plot=FALSE, nSim=10000)

#Outbreak 5
WT5_3.5 <- WT(outbreak5, T.Start=1:12, T.End=1:12, method="ParametricSI", Mean.SI=3.5, Std.SI=1.998, plot=FALSE, nSim=10000)

#Outbreak 6
WT6_3.5 <- WT(outbreak6, T.Start=1:12, T.End=1:12, method="ParametricSI", Mean.SI=3.5, Std.SI=1.998, plot=FALSE, nSim=10000)

##################################### Mean Serial Interval of 4.0 Days ##################################### 
#Outbreak 1
WT1_4.0 <- WT(outbreak1, T.Start=1:12, T.End=1:12, method="ParametricSI", Mean.SI=4.0, Std.SI=1.998, plot=FALSE, nSim=10000)

#Outbreak 2
WT2_4.0 <- WT(outbreak2, T.Start=1:10, T.End=1:10, method="ParametricSI", Mean.SI=4.0, Std.SI=1.998, plot=FALSE, nSim=10000)
```

```
## Warning in WT(outbreak2, T.Start = 1:10, T.End = 1:10, method = "ParametricSI", : The serial interval distribution you have chosen is very wide compared to the duration of the epidemic.
## Estimation will be performed anyway but restults should be interpreted with care.
```

```
#Outbreak 3
WT3_4.0 <- WT(outbreak3, T.Start=1:13, T.End=1:13, method="ParametricSI", Mean.SI=4.0, Std.SI=1.998, plot=FALSE, nSim=10000)

#Outbreak 4
WT4_4.0 <- WT(outbreak4, T.Start=1:18, T.End=1:18, method="ParametricSI", Mean.SI=4.0, Std.SI=1.998, plot=FALSE, nSim=10000)

#Outbreak 5
WT5_4.0 <- WT(outbreak5, T.Start=1:12, T.End=1:12, method="ParametricSI", Mean.SI=4.0, Std.SI=1.998, plot=FALSE, nSim=10000)

#Outbreak 6
WT6_4.0 <- WT(outbreak6, T.Start=1:12, T.End=1:12, method="ParametricSI", Mean.SI=4.0, Std.SI=1.998, plot=FALSE, nSim=10000)
```

Next, save the estimated reproduction numbers for each outbreak and each mean serial interval length as separate CSV files.

```
##################################### Mean Serial Interval of 1.5 Days ##################################### 

#Outbreak 1
write.csv(cbind(WT1_1.5$R$`Mean(R)`, (WT1_1.5$R$`Std(R)`)^2), "outbreak1_1.5.csv")

#Outbreak 2
write.csv(cbind(WT2_1.5$R$`Mean(R)`, (WT2_1.5$R$`Std(R)`)^2), "outbreak2_1.5.csv")

#Outbreak 3
write.csv(cbind(WT3_1.5$R$`Mean(R)`, (WT3_1.5$R$`Std(R)`)^2), "outbreak3_1.5.csv")

#Outbreak 4
write.csv(cbind(WT4_1.5$R$`Mean(R)`, (WT4_1.5$R$`Std(R)`)^2), "outbreak4_1.5.csv")

#Outbreak 5
write.csv(cbind(WT5_1.5$R$`Mean(R)`, (WT5_1.5$R$`Std(R)`)^2), "outbreak5_1.5.csv")

#Outbreak 6
write.csv(cbind(WT6_1.5$R$`Mean(R)`, (WT6_1.5$R$`Std(R)`)^2), "outbreak6_1.5.csv")


##################################### Mean Serial Interval of 2.0 Days ##################################### 
#Outbreak 1
write.csv(cbind(WT1_2.0$R$`Mean(R)`,(WT1_2.0$R$`Std(R)`)^2), "outbreak1_2.0.csv")

#Outbreak 2
write.csv(cbind(WT2_2.0$R$`Mean(R)`,(WT2_2.0$R$`Std(R)`)^2), "outbreak2_2.0.csv")

#Outbreak 3
write.csv(cbind(WT3_2.0$R$`Mean(R)`,(WT3_2.0$R$`Std(R)`)^2), "outbreak3_2.0.csv")

#Outbreak 4
write.csv(cbind(WT4_2.0$R$`Mean(R)`,(WT4_2.0$R$`Std(R)`)^2), "outbreak4_2.0.csv")

#Outbreak 5
write.csv(cbind(WT5_2.0$R$`Mean(R)`,(WT5_2.0$R$`Std(R)`)^2), "outbreak5_2.0.csv")

#Outbreak 6
write.csv(cbind(WT6_2.0$R$`Mean(R)`,(WT6_2.0$R$`Std(R)`)^2), "outbreak6_2.0.csv")


##################################### Mean Serial Interval of 2.5 Days ##################################### 
#Outbreak 1
write.csv(cbind(WT1_2.5$R$`Mean(R)`,(WT1_2.5$R$`Std(R)`)^2), "outbreak1_2.5.csv")

#Outbreak 2
write.csv(cbind(WT2_2.5$R$`Mean(R)`,(WT2_2.5$R$`Std(R)`)^2), "outbreak2_2.5.csv")

#Outbreak 3
write.csv(cbind(WT3_2.5$R$`Mean(R)`,(WT3_2.5$R$`Std(R)`)^2), "outbreak3_2.5.csv")

#Outbreak 4
write.csv(cbind(WT4_2.5$R$`Mean(R)`,(WT4_2.5$R$`Std(R)`)^2), "outbreak4_2.5.csv")

#Outbreak 5
write.csv(cbind(WT5_2.5$R$`Mean(R)`,(WT5_2.5$R$`Std(R)`)^2), "outbreak5_2.5.csv")

#Outbreak 6
write.csv(cbind(WT6_2.5$R$`Mean(R)`,(WT6_2.5$R$`Std(R)`)^2), "outbreak6_2.5.csv")


##################################### Mean Serial Interval of 3.0 Days ##################################### 
#Outbreak 1
write.csv(cbind(WT1_3.0$R$`Mean(R)`,(WT1_3.0$R$`Std(R)`)^2), "outbreak1_3.0.csv")

#Outbreak 2
write.csv(cbind(WT2_3.0$R$`Mean(R)`,(WT2_3.0$R$`Std(R)`)^2), "outbreak2_3.0.csv")

#Outbreak 3
write.csv(cbind(WT3_3.0$R$`Mean(R)`,(WT3_3.0$R$`Std(R)`)^2), "outbreak3_3.0.csv")

#Outbreak 4
write.csv(cbind(WT4_3.0$R$`Mean(R)`,(WT4_3.0$R$`Std(R)`)^2), "outbreak4_3.0.csv")

#Outbreak 5
write.csv(cbind(WT5_3.0$R$`Mean(R)`,(WT5_3.0$R$`Std(R)`)^2), "outbreak5_3.0.csv")

#Outbreak 6
write.csv(cbind(WT6_3.0$R$`Mean(R)`,(WT6_3.0$R$`Std(R)`)^2), "outbreak6_3.0.csv")


##################################### Mean Serial Interval of 3.5 Days ##################################### 
#Outbreak 1
write.csv(cbind(WT1_3.5$R$`Mean(R)`,(WT1_3.5$R$`Std(R)`)^2), "outbreak1_3.5.csv")

#Outbreak 2
write.csv(cbind(WT2_3.5$R$`Mean(R)`,(WT2_3.5$R$`Std(R)`)^2), "outbreak2_3.5.csv")

#Outbreak 3
write.csv(cbind(WT3_3.5$R$`Mean(R)`,(WT3_3.5$R$`Std(R)`)^2), "outbreak3_3.5.csv")

#Outbreak 4
write.csv(cbind(WT4_3.5$R$`Mean(R)`,(WT4_3.5$R$`Std(R)`)^2), "outbreak4_3.5.csv")

#Outbreak 5
write.csv(cbind(WT5_3.5$R$`Mean(R)`,(WT5_3.5$R$`Std(R)`)^2), "outbreak5_3.5.csv")

#Outbreak 6
write.csv(cbind(WT6_3.5$R$`Mean(R)`,(WT6_3.5$R$`Std(R)`)^2), "outbreak6_3.5.csv")


##################################### Mean Serial Interval of 4.0 Days ##################################### 
#Outbreak 1
write.csv(cbind(WT1_4.0$R$`Mean(R)`, (WT1_4.0$R$`Std(R)`)^2), "outbreak1_4.0.csv")

#Outbreak 2
write.csv(cbind(WT2_4.0$R$`Mean(R)`, (WT2_4.0$R$`Std(R)`)^2), "outbreak2_4.0.csv")

#Outbreak 3
write.csv(cbind(WT3_4.0$R$`Mean(R)`, (WT3_4.0$R$`Std(R)`)^2), "outbreak3_4.0.csv")

#Outbreak 4
write.csv(cbind(WT4_4.0$R$`Mean(R)`, (WT4_4.0$R$`Std(R)`)^2), "outbreak4_4.0.csv")

#Outbreak 5
write.csv(cbind(WT5_4.0$R$`Mean(R)`, (WT5_4.0$R$`Std(R)`)^2), "outbreak5_4.0.csv")

#Outbreak 6
write.csv(cbind(WT6_4.0$R$`Mean(R)`, (WT6_4.0$R$`Std(R)`)^2), "outbreak6_4.0.csv")
```

The last step is to assign reproduction numbers, 95% CIs, and variances to all cases involved in the 6 outbreaks for each serial interval length. This has already been done for you in the provided file: “S1\_File.csv”. However, were you to do this yourself, you would open the CSV files created for each outbreak and each serial interval length and also open the combined line list with cases from all outbreaks (“S1\_File.csv”). You would create twelve new columns in S1\_File.csv, one column for the reproduction number estimates and one column for the variances (the standard deviation squared) for each of the six serial interval lengths (1.5, 2.0, 2.5, 3.0, 3.5, and 4.0). You would then manually copy and paste the estimated reproduction numbers and variances for each serial interval length into the rows corresponding to the correct outbreak and onset day. Cases with the same days of illness onset, within a given outbreak, would be assigned identical reproduction numbers and variances.
