# Supplementary material for: Quantifying the roles of vomiting, diarrhea, and residents vs. staff in norovirus transmission in U.S. nursing home outbreaks
Source: PLoS Comput Biol. 2020 Mar 25;16(3):e1007271. doi: 10.1371/journal.pcbi.1007271 (PMC7135310; doi:10.1371/journal.pcbi.1007271)
Supplement: S3 RMarkdown — This file contains instuctions and R code for running weighted mixed regression analyses. (HTML) [file pcbi.1007271.s004.html]

Mixed linear regression analysis: weighted


# Mixed linear regression analysis: weighted

#### *Carly Adams*

Set your working directory, import the necessary dataset, and load the necessary packages.

```
setwd("C:/Users/cladam3/OneDrive - Emory University/Desktop/Norovirus_RA/Manuscript Related/PLOS/Excel Calculations/Excel")
outbreaks <- read.csv("outbreaks_all.csv")

library(metafor)
```

```
## Loading required package: Matrix
```

```
## Loading 'metafor' package (version 2.0-0). For an overview 
## and introduction to the package please type: help(metafor).
```

```
library(expss)
```

```
## Warning: package 'expss' was built under R version 3.5.3
```

```
## 
## Use 'expss_output_rnotebook()' to display tables inside R Notebooks.
##  To return to the console output, use 'expss_output_default()'.
```

```
library(dplyr)
```

```
## 
## Attaching package: 'dplyr'
```

```
## The following objects are masked from 'package:expss':
## 
##     between, compute, contains, first, last, na_if, recode, vars
```

```
## The following objects are masked from 'package:stats':
## 
##     filter, lag
```

```
## The following objects are masked from 'package:base':
## 
##     intersect, setdiff, setequal, union
```

```
library(lme4)
```

```
## 
## Attaching package: 'lme4'
```

```
## The following object is masked from 'package:expss':
## 
##     dummy
```

Main Analysis:

First, exclude the one case missing an onset date (and therefore an REi estimate).

```
outbreaks <- outbreaks[!is.na(outbreaks$R),]  #Excluding the one case without an onset date
```

Second, calculate the within-outbreak variance. To do this, use the dplyr package to group observations by outbreak and then find the variance of REi estimates within each outbreak.

```
w <- outbreaks %>% 
  group_by(Outbreak_Number) %>% 
  summarise(count=n(), within.var = var(R)) 
w
```

```
## # A tibble: 6 x 3
##   Outbreak_Number count within.var
##             <int> <int>      <dbl>
## 1               1    27      1.78 
## 2               2    11      0.272
## 3               3    46      2.51 
## 4               4    51      3.63 
## 5               5    32      1.40 
## 6               6    41      2.02
```

Third, calculate the between-outbreak variance. To do this, use ANOVA to calculate the variance between mean REi values for each outbreak. This variance is then equal to the sum of squares divided by the degrees of freedom.

```
b <- anova(lm(R ~ factor(Outbreak_Number), outbreaks))
between.var = b$`Sum Sq`[1]/b$Df[1]
between.var
```

```
## [1] 0.1291716
```

Fourth, create the dataset to be used for the weighted regression. Start by removing observations with missing outcome values. Then, calculate the number of observations for each outbreak and sort the dataset to ensure it is in order of outbreak number and case number. Next, create the within-variance variable by repeating each outbreak’s within-variance by the number of observations in that outbreak. Create the between-variance variable by repeating the between-variance by the total number of observations. Lastly, create the weight variable by taking the inverse of the sum of the three variance components.

```
meta.data <- outbreaks[!is.na(outbreaks$logR),]

count.outbreak1 <- count_if("1", meta.data$Outbreak_Number)
count.outbreak2 <- count_if("2", meta.data$Outbreak_Number)
count.outbreak3 <- count_if("3", meta.data$Outbreak_Number)
count.outbreak4 <- count_if("4", meta.data$Outbreak_Number)
count.outbreak5 <- count_if("5", meta.data$Outbreak_Number)
count.outbreak6 <- count_if("6", meta.data$Outbreak_Number)
count.all <- nrow(meta.data)

meta.data <- meta.data[order(meta.data$Onset_Day),]        
meta.data <- meta.data[order(meta.data$Outbreak_Number),] 

meta.data$within.var <- rep(as.numeric(c(w[1,3], w[2,3], w[3,3], w[4,3], w[5,3], w[6,3])), 
    times = c(count.outbreak1, count.outbreak2, count.outbreak3, count.outbreak4, count.outbreak5, count.outbreak6))

meta.data$between.var <- rep(as.numeric(between.var), count.all)

meta.data$weight <- 1/(meta.data$var_R + meta.data$within.var + meta.data$between.var)
```

Fifth, name variables and convert some variables to numeric or factors.

```
logR <- meta.data$logR
logR_1.5 <- as.numeric(as.character(meta.data$log_R_1.5))
logR_2.0 <- as.numeric(as.character(meta.data$log_R_2.0))
logR_2.5 <- as.numeric(as.character(meta.data$log_R_2.5))
logR_3.0 <- as.numeric(as.character(meta.data$log_R_3.0))
logR_3.5 <- as.numeric(as.character(meta.data$log_R_3.5))
logR_4.0 <- as.numeric(as.character(meta.data$log_R_4.0))
resident <- meta.data$Resident
diarrhea <- meta.data$Diarrhea
vomit <- meta.data$Vomit
outbreak.num <- factor(meta.data$Outbreak_Number)
var.R <- meta.data$var_R
wei <- meta.data$weight
index <- meta.data$Index
onset <- meta.data$Onset_Day
age <- meta.data$Age
sex <- meta.data$Female
```

Sixth, use the metafor package and the rma.mv function to run the mixed meta-regression analysis. Set the outcome to log REi and the variance to that of the variance of the REi estimates plus 0.01. Adding 0.01 does not change coefficient or uncertainty estimates (including an argument for weights, W, overrides the V argument), but ensures that the model will run. Otherwise, there are “non-positive” var REi estimates (REi = 0) and the model will not run. Include weights in the model by setting W = wei, which specifies that weights should be equal to the calculated weight for each REi estimates (the inverse of the sum of the 3 variances). Specify the variance structure as unknown. Next, include the moderators (the independent variables): resident, diarrhea, and vomit. Specify that the model is a restricted maximum-likelihood estimator (REML). Lastly, indicate that there is a random intercept for outbreak.

```
mv_r1 <- rma.mv(yi=logR, V=var.R + 0.01, W=wei, struct="UN",
                mods= ~ resident + diarrhea + vomit, method="REML", random = ~ 1 | outbreak.num)
```

```
## Warning in rma.mv(yi = logR, V = var.R + 0.01, W = wei, struct = "UN", mods
## = ~resident + : Rows with NAs omitted from model fitting.
```

```
mv_r1
```

```
## 
## Multivariate Meta-Analysis Model (k = 195; method: REML)
## 
## Variance Components: 
## 
##             estim    sqrt  nlvls  fixed        factor
## sigma^2    0.0811  0.2847      6     no  outbreak.num
## 
## Test for Residual Heterogeneity: 
## QE(df = 191) = 3390.3636, p-val < .0001
## 
## Test of Moderators (coefficient(s) 2:4): 
## QM(df = 3) = 42.7777, p-val < .0001
## 
## Model Results:
## 
##           estimate      se     zval    pval    ci.lb    ci.ub     
## intrcpt    -1.7592  0.2801  -6.2808  <.0001  -2.3081  -1.2102  ***
## resident    0.4241  0.1429   2.9684  0.0030   0.1441   0.7041   **
## diarrhea    0.3284  0.1528   2.1491  0.0316   0.0289   0.6279    *
## vomit       0.7508  0.1195   6.2804  <.0001   0.5165   0.9851  ***
## 
## ---
## Signif. codes:  0 '***' 0.001 '**' 0.01 '*' 0.05 '.' 0.1 ' ' 1
```

Seventh, exponentiate the regression coefficients and corresponding confidence intervals to calculae results from the main regression analysis.

```
mv_r1_exp <- cbind(exp(mv_r1$beta), exp(mv_r1$ci.lb), exp(mv_r1$ci.ub))
mv_r1_exp
```

```
##               [,1]       [,2]      [,3]
## intrcpt  0.1721889 0.09944802 0.2981358
## resident 1.5282406 1.15498388 2.0221230
## diarrhea 1.3887420 1.02932472 1.8736597
## vomit    2.1186538 1.67611461 2.6780352
```

Additional Analyses:

1. Determining relative infectiousness of index cases:

Include the dichotomous variable for index cases to examine the relative infectiousness of index cases compared to non-index cases. Run the same model above including the dichotomous variable for index cases and exponentiate results.

```
mv_r2 <- rma.mv(yi=logR, V=var.R + 0.01, W=wei, struct="UN", 
                mods= ~ resident + diarrhea + vomit + index, method="REML", random = ~ 1 | outbreak.num)
```

```
## Warning in rma.mv(yi = logR, V = var.R + 0.01, W = wei, struct = "UN", mods
## = ~resident + : Rows with NAs omitted from model fitting.
```

```
mv_r2
```

```
## 
## Multivariate Meta-Analysis Model (k = 195; method: REML)
## 
## Variance Components: 
## 
##             estim    sqrt  nlvls  fixed        factor
## sigma^2    0.0561  0.2368      6     no  outbreak.num
## 
## Test for Residual Heterogeneity: 
## QE(df = 190) = 3297.1556, p-val < .0001
## 
## Test of Moderators (coefficient(s) 2:5): 
## QM(df = 4) = 77.7794, p-val < .0001
## 
## Model Results:
## 
##           estimate      se     zval    pval    ci.lb    ci.ub     
## intrcpt    -1.6033  0.2396  -6.6923  <.0001  -2.0729  -1.1337  ***
## resident    0.2905  0.1169   2.4855  0.0129   0.0614   0.5196    *
## diarrhea    0.1724  0.1443   1.1949  0.2321  -0.1104   0.4552     
## vomit       0.6844  0.1039   6.5885  <.0001   0.4808   0.8879  ***
## index       1.3767  0.2478   5.5553  <.0001   0.8910   1.8624  ***
## 
## ---
## Signif. codes:  0 '***' 0.001 '**' 0.01 '*' 0.05 '.' 0.1 ' ' 1
```

```
mv_r2_exp <- cbind(exp(mv_r2$beta), exp(mv_r2$ci.lb), exp(mv_r2$ci.ub))
mv_r2_exp
```

```
##               [,1]      [,2]      [,3]
## intrcpt  0.2012309 0.1258254 0.3218258
## resident 1.3370941 1.0633526 1.6813055
## diarrhea 1.1881423 0.8955010 1.5764159
## vomit    1.9825044 1.6173295 2.4301317
## index    3.9617777 2.4375124 6.4392216
```

2. Examining associations between exposure variables and onset day:

Examine the association between onset day and case characteristics to examine if cases who vomit, have diarrhea, and are residents occur earlier in the outbreak. To do this, use a non-weighted mixed linear regression model with outbreak day as the outcome variable.

```
mixed.onset <- lmer(onset ~ resident + diarrhea + vomit + (1|outbreak.num))
summary(mixed.onset)
```

```
## Linear mixed model fit by REML ['lmerMod']
## Formula: onset ~ resident + diarrhea + vomit + (1 | outbreak.num)
## 
## REML criterion at convergence: 861.3
## 
## Scaled residuals: 
##     Min      1Q  Median      3Q     Max 
## -3.6797 -0.4280  0.0487  0.4884  3.1999 
## 
## Random effects:
##  Groups       Name        Variance Std.Dev.
##  outbreak.num (Intercept) 5.117    2.262   
##  Residual                 4.479    2.116   
## Number of obs: 195, groups:  outbreak.num, 6
## 
## Fixed effects:
##             Estimate Std. Error t value
## (Intercept)  10.8504     1.1620   9.338
## resident     -1.5827     0.3937  -4.020
## diarrhea     -1.7617     0.4481  -3.932
## vomit        -2.6823     0.4019  -6.674
## 
## Correlation of Fixed Effects:
##          (Intr) resdnt diarrh
## resident -0.384              
## diarrhea -0.437  0.162       
## vomit    -0.439  0.257  0.371
```

```
confint(mixed.onset)
```

```
## Computing profile confidence intervals ...
```

```
##                 2.5 %     97.5 %
## .sig01       1.224991  4.1915420
## .sigma       1.904425  2.3304773
## (Intercept)  8.550734 13.1797324
## resident    -2.347647 -0.8083069
## diarrhea    -2.628223 -0.8711701
## vomit       -3.462457 -1.8903118
```

Sensitivity Analyses:

1. Changing serial interval lengths:

Examine the association between REi and case characteristics using REi estimates that were calculated assuming different average serial interval lengths (1.5, 2.0, 2.5, 3.0, 3.5, and 4.5 days). We’ll run the same model as above but use REi estimates that were calculated assuming these different average serial interval lengths, and then exponentiate results.

```
#Assuming mean SI = 1.5
mv_1.5 <- rma.mv(yi=logR_1.5, V= var.R + 0.01, 
                   W=1/(meta.data$var_R_1.5 + meta.data$between.var + meta.data$within.var), 
                   struct="UN", 
                   mods= ~ resident + diarrhea + vomit, method="REML", random = ~ 1 | outbreak.num)
```

```
## Warning in rma.mv(yi = logR_1.5, V = var.R + 0.01, W = 1/(meta.data
## $var_R_1.5 + : Rows with NAs omitted from model fitting.
```

```
mv_1.5
```

```
## 
## Multivariate Meta-Analysis Model (k = 195; method: REML)
## 
## Variance Components: 
## 
##             estim    sqrt  nlvls  fixed        factor
## sigma^2    0.0286  0.1690      6     no  outbreak.num
## 
## Test for Residual Heterogeneity: 
## QE(df = 191) = 1668.0151, p-val < .0001
## 
## Test of Moderators (coefficient(s) 2:4): 
## QM(df = 3) = 15.9701, p-val = 0.0012
## 
## Model Results:
## 
##           estimate      se     zval    pval    ci.lb    ci.ub     
## intrcpt    -1.0140  0.1894  -5.3545  <.0001  -1.3851  -0.6428  ***
## resident    0.1068  0.1011   1.0567  0.2906  -0.0913   0.3049     
## diarrhea    0.4121  0.1209   3.4072  0.0007   0.1750   0.6491  ***
## vomit       0.2836  0.0905   3.1350  0.0017   0.1063   0.4610   **
## 
## ---
## Signif. codes:  0 '***' 0.001 '**' 0.01 '*' 0.05 '.' 0.1 ' ' 1
```

```
mv_1.5_exp <- cbind(exp(mv_1.5$beta), exp(mv_1.5$ci.lb), exp(mv_1.5$ci.ub))
mv_1.5_exp
```

```
##               [,1]      [,2]      [,3]
## intrcpt  0.3627789 0.2502956 0.5258122
## resident 1.1127075 0.9127575 1.3564589
## diarrhea 1.5099458 1.1912843 1.9138473
## vomit    1.3279497 1.1121681 1.5855970
```

```
#Assuming mean SI = 2.0
mv_2.0 <- rma.mv(yi=logR_2.0, V= var.R + 0.01, 
                   W=1/(meta.data$var_R_2.0 + meta.data$between.var + meta.data$within.var), 
                   struct="UN", 
                   mods= ~ resident + diarrhea + vomit, method="REML", random = ~ 1 | outbreak.num)
```

```
## Warning in rma.mv(yi = logR_2.0, V = var.R + 0.01, W = 1/(meta.data
## $var_R_2.0 + : Rows with NAs omitted from model fitting.
```

```
mv_2.0
```

```
## 
## Multivariate Meta-Analysis Model (k = 195; method: REML)
## 
## Variance Components: 
## 
##             estim    sqrt  nlvls  fixed        factor
## sigma^2    0.0253  0.1590      6     no  outbreak.num
## 
## Test for Residual Heterogeneity: 
## QE(df = 191) = 1396.0241, p-val < .0001
## 
## Test of Moderators (coefficient(s) 2:4): 
## QM(df = 3) = 18.9859, p-val = 0.0003
## 
## Model Results:
## 
##           estimate      se     zval    pval    ci.lb    ci.ub     
## intrcpt    -1.0522  0.1808  -5.8202  <.0001  -1.4066  -0.6979  ***
## resident    0.1753  0.0957   1.8324  0.0669  -0.0122   0.3628    .
## diarrhea    0.3576  0.1148   3.1164  0.0018   0.1327   0.5825   **
## vomit       0.3463  0.0869   3.9868  <.0001   0.1760   0.5165  ***
## 
## ---
## Signif. codes:  0 '***' 0.001 '**' 0.01 '*' 0.05 '.' 0.1 ' ' 1
```

```
mv_2.0_exp <- cbind(exp(mv_2.0$beta), exp(mv_2.0$ci.lb), exp(mv_2.0$ci.ub))
mv_2.0_exp
```

```
##               [,1]      [,2]      [,3]
## intrcpt  0.3491577 0.2449812 0.4976344
## resident 1.1916145 0.9878727 1.4373766
## diarrhea 1.4299091 1.1419119 1.7905409
## vomit    1.4137897 1.1924878 1.6761609
```

```
#Assuming mean SI = 2.5
mv_2.5 <- rma.mv(yi=logR_2.5, V= var.R + 0.01, 
                   W=1/(meta.data$var_R_2.5 + meta.data$between.var + meta.data$within.var), 
                   struct="UN", 
                   mods= ~ resident + diarrhea + vomit, method="REML", random = ~ 1 | outbreak.num)
```

```
## Warning in rma.mv(yi = logR_2.5, V = var.R + 0.01, W = 1/(meta.data
## $var_R_2.5 + : Rows with NAs omitted from model fitting.
```

```
mv_2.5
```

```
## 
## Multivariate Meta-Analysis Model (k = 195; method: REML)
## 
## Variance Components: 
## 
##             estim    sqrt  nlvls  fixed        factor
## sigma^2    0.0305  0.1745      6     no  outbreak.num
## 
## Test for Residual Heterogeneity: 
## QE(df = 191) = 1659.4286, p-val < .0001
## 
## Test of Moderators (coefficient(s) 2:4): 
## QM(df = 3) = 25.1638, p-val < .0001
## 
## Model Results:
## 
##           estimate      se     zval    pval    ci.lb    ci.ub     
## intrcpt    -1.1980  0.1915  -6.2560  <.0001  -1.5733  -0.8227  ***
## resident    0.2630  0.0999   2.6329  0.0085   0.0672   0.4589   **
## diarrhea    0.3217  0.1169   2.7510  0.0059   0.0925   0.5509   **
## vomit       0.4431  0.0897   4.9407  <.0001   0.2673   0.6189  ***
## 
## ---
## Signif. codes:  0 '***' 0.001 '**' 0.01 '*' 0.05 '.' 0.1 ' ' 1
```

```
mv_2.5_exp <- cbind(exp(mv_2.5$beta), exp(mv_2.5$ci.lb), exp(mv_2.5$ci.ub))
mv_2.5_exp
```

```
##               [,1]      [,2]      [,3]
## intrcpt  0.3017919 0.2073498 0.4392498
## resident 1.3008809 1.0695378 1.5822639
## diarrhea 1.3794835 1.0969188 1.7348365
## vomit    1.5575542 1.3064760 1.8568845
```

```
#Assuming mean SI = 3.0
mv_3.0 <- rma.mv(yi=logR_3.0, V= var.R + 0.01, 
                   W=1/(meta.data$var_R_3.0 + meta.data$between.var + meta.data$within.var), 
                   struct="UN", 
                   mods= ~ resident + diarrhea + vomit, method="REML", random = ~ 1 | outbreak.num)
```

```
## Warning in rma.mv(yi = logR_3.0, V = var.R + 0.01, W = 1/(meta.data
## $var_R_3.0 + : Rows with NAs omitted from model fitting.
```

```
mv_3.0
```

```
## 
## Multivariate Meta-Analysis Model (k = 195; method: REML)
## 
## Variance Components: 
## 
##             estim    sqrt  nlvls  fixed        factor
## sigma^2    0.0441  0.2099      6     no  outbreak.num
## 
## Test for Residual Heterogeneity: 
## QE(df = 191) = 2218.9093, p-val < .0001
## 
## Test of Moderators (coefficient(s) 2:4): 
## QM(df = 3) = 33.3833, p-val < .0001
## 
## Model Results:
## 
##           estimate      se     zval    pval    ci.lb    ci.ub     
## intrcpt    -1.4065  0.2186  -6.4331  <.0001  -1.8350  -0.9780  ***
## resident    0.3377  0.1125   3.0025  0.0027   0.1172   0.5581   **
## diarrhea    0.3082  0.1268   2.4309  0.0151   0.0597   0.5566    *
## vomit       0.5652  0.0982   5.7563  <.0001   0.3728   0.7577  ***
## 
## ---
## Signif. codes:  0 '***' 0.001 '**' 0.01 '*' 0.05 '.' 0.1 ' ' 1
```

```
mv_3.0_exp <- cbind(exp(mv_3.0$beta), exp(mv_3.0$ci.lb), exp(mv_3.0$ci.ub))
mv_3.0_exp
```

```
##               [,1]      [,2]      [,3]
## intrcpt  0.2450061 0.1596165 0.3760765
## resident 1.4016552 1.1243892 1.7472930
## diarrhea 1.3609257 1.0615139 1.7447899
## vomit    1.7598802 1.4517701 2.1333808
```

```
#Assuming mean SI = 3.5
mv_3.5 <- rma.mv(yi=logR_3.5, V= var.R + 0.01, 
                   W=1/(meta.data$var_R_3.5 + meta.data$between.var + meta.data$within.var), 
                   struct="UN", 
                   mods= ~ resident + diarrhea + vomit, method="REML", random = ~ 1 | outbreak.num)
```

```
## Warning in rma.mv(yi = logR_3.5, V = var.R + 0.01, W = 1/(meta.data
## $var_R_3.5 + : Rows with NAs omitted from model fitting.
```

```
mv_3.5
```

```
## 
## Multivariate Meta-Analysis Model (k = 195; method: REML)
## 
## Variance Components: 
## 
##             estim    sqrt  nlvls  fixed        factor
## sigma^2    0.0682  0.2612      6     no  outbreak.num
## 
## Test for Residual Heterogeneity: 
## QE(df = 191) = 3117.6859, p-val < .0001
## 
## Test of Moderators (coefficient(s) 2:4): 
## QM(df = 3) = 41.9525, p-val < .0001
## 
## Model Results:
## 
##           estimate      se     zval    pval    ci.lb    ci.ub     
## intrcpt    -1.7020  0.2603  -6.5374  <.0001  -2.2123  -1.1917  ***
## resident    0.4219  0.1330   3.1717  0.0015   0.1612   0.6825   **
## diarrhea    0.3276  0.1442   2.2715  0.0231   0.0449   0.6102    *
## vomit       0.7120  0.1125   6.3311  <.0001   0.4916   0.9325  ***
## 
## ---
## Signif. codes:  0 '***' 0.001 '**' 0.01 '*' 0.05 '.' 0.1 ' ' 1
```

```
mv_3.5_exp <- cbind(exp(mv_3.5$beta), exp(mv_3.5$ci.lb), exp(mv_3.5$ci.ub))
mv_3.5_exp
```

```
##               [,1]      [,2]      [,3]
## intrcpt  0.1823186 0.1094519 0.3036957
## resident 1.5247809 1.1748837 1.9788823
## diarrhea 1.3875780 1.0459445 1.8407980
## vomit    2.0381271 1.6349365 2.5407482
```

```
#Assuming mean SI = 4.0
mv_4.0 <- rma.mv(yi=logR_4.0, V= var.R + 0.01, 
                   W=1/(meta.data$var_R_4.0 + meta.data$between.var + meta.data$within.var), 
                   struct="UN", 
                   mods= ~ resident + diarrhea + vomit, method="REML", random = ~ 1 | outbreak.num)
```

```
## Warning in rma.mv(yi = logR_4.0, V = var.R + 0.01, W = 1/(meta.data
## $var_R_4.0 + : Rows with NAs omitted from model fitting.
```

```
mv_4.0
```

```
## 
## Multivariate Meta-Analysis Model (k = 195; method: REML)
## 
## Variance Components: 
## 
##             estim    sqrt  nlvls  fixed        factor
## sigma^2    0.1241  0.3522      6     no  outbreak.num
## 
## Test for Residual Heterogeneity: 
## QE(df = 191) = 4311.6929, p-val < .0001
## 
## Test of Moderators (coefficient(s) 2:4): 
## QM(df = 3) = 46.9274, p-val < .0001
## 
## Model Results:
## 
##           estimate      se     zval    pval    ci.lb    ci.ub     
## intrcpt    -2.0488  0.3377  -6.0662  <.0001  -2.7107  -1.3868  ***
## resident    0.4807  0.1718   2.7981  0.0051   0.1440   0.8174   **
## diarrhea    0.3687  0.1786   2.0650  0.0389   0.0187   0.7187    *
## vomit       0.8893  0.1405   6.3293  <.0001   0.6139   1.1647  ***
## 
## ---
## Signif. codes:  0 '***' 0.001 '**' 0.01 '*' 0.05 '.' 0.1 ' ' 1
```

```
mv_4.0_exp <- cbind(exp(mv_4.0$beta), exp(mv_4.0$ci.lb), exp(mv_4.0$ci.ub))
mv_4.0_exp
```

```
##               [,1]       [,2]      [,3]
## intrcpt  0.1288925 0.06648856 0.2498665
## resident 1.6172316 1.15487817 2.2646875
## diarrhea 1.4458616 1.01892349 2.0516906
## vomit    2.4335146 1.84769736 3.2050666
```

2. Adding age and/or sex to the model:

Examine confounding by sex and age for the 113 cases who had this information. To do this, we will include sex and/or age in the regression model to examine whether or not coefficient estimates meaningfully change. We will also examine associations between sex and age and the outcome (log REi) and exposures (vomit, diarrhea, and resident)

```
# First, running the mixed model including sex
mv_sex <- rma.mv(yi=logR, V=var.R + 0.01, W=wei, struct="UN", 
                mods= ~ resident + diarrhea + vomit + sex, method="REML", random = ~ 1 | outbreak.num)
```

```
## Warning in rma.mv(yi = logR, V = var.R + 0.01, W = wei, struct = "UN", mods
## = ~resident + : Rows with NAs omitted from model fitting.
```

```
mv_sex
```

```
## 
## Multivariate Meta-Analysis Model (k = 145; method: REML)
## 
## Variance Components: 
## 
##             estim    sqrt  nlvls  fixed        factor
## sigma^2    0.1203  0.3469      5     no  outbreak.num
## 
## Test for Residual Heterogeneity: 
## QE(df = 140) = 2426.2236, p-val < .0001
## 
## Test of Moderators (coefficient(s) 2:5): 
## QM(df = 4) = 24.1531, p-val < .0001
## 
## Model Results:
## 
##           estimate      se     zval    pval    ci.lb    ci.ub     
## intrcpt    -1.0763  0.3803  -2.8306  0.0046  -1.8216  -0.3310   **
## resident    0.1198  0.2087   0.5742  0.5658  -0.2892   0.5288     
## diarrhea    0.1119  0.1652   0.6776  0.4980  -0.2119   0.4357     
## vomit       0.5588  0.1610   3.4703  0.0005   0.2432   0.8744  ***
## sex        -0.0438  0.1004  -0.4361  0.6628  -0.2405   0.1529     
## 
## ---
## Signif. codes:  0 '***' 0.001 '**' 0.01 '*' 0.05 '.' 0.1 ' ' 1
```

```
mv_sex_exp <- cbind(exp(mv_sex$beta), exp(mv_sex$ci.lb), exp(mv_sex$ci.ub))
mv_sex_exp
```

```
##               [,1]      [,2]      [,3]
## intrcpt  0.3408421 0.1617631 0.7181695
## resident 1.1273082 0.7488857 1.6969528
## diarrhea 1.1184558 0.8090860 1.5461190
## vomit    1.7485301 1.2753100 2.3973445
## sex      0.9571721 0.7862427 1.1652615
```

```
# Second, running the mixed model including age
mv_age <- rma.mv(yi=logR, V=var.R + 0.01, W=wei, struct="UN", 
                mods= ~ resident + diarrhea + vomit + age, method="REML", random = ~ 1 | outbreak.num)
```

```
## Warning in rma.mv(yi = logR, V = var.R + 0.01, W = wei, struct = "UN", mods
## = ~resident + : Rows with NAs omitted from model fitting.
```

```
mv_age
```

```
## 
## Multivariate Meta-Analysis Model (k = 147; method: REML)
## 
## Variance Components: 
## 
##             estim    sqrt  nlvls  fixed        factor
## sigma^2    0.0910  0.3017      6     no  outbreak.num
## 
## Test for Residual Heterogeneity: 
## QE(df = 142) = 2621.5112, p-val < .0001
## 
## Test of Moderators (coefficient(s) 2:5): 
## QM(df = 4) = 41.4511, p-val < .0001
## 
## Model Results:
## 
##           estimate      se     zval    pval    ci.lb    ci.ub     
## intrcpt    -1.7355  0.3705  -4.6838  <.0001  -2.4617  -1.0093  ***
## resident   -0.2133  0.3602  -0.5924  0.5536  -0.9192   0.4925     
## diarrhea    0.1571  0.1746   0.9000  0.3681  -0.1851   0.4993     
## vomit       0.8071  0.1340   6.0256  <.0001   0.5446   1.0697  ***
## age         0.0086  0.0055   1.5673  0.1170  -0.0022   0.0194     
## 
## ---
## Signif. codes:  0 '***' 0.001 '**' 0.01 '*' 0.05 '.' 0.1 ' ' 1
```

```
mv_age_exp <- cbind(exp(mv_age$beta), exp(mv_age$ci.lb), exp(mv_age$ci.ub))
mv_age_exp
```

```
##               [,1]       [,2]      [,3]
## intrcpt  0.1763141 0.08528863 0.3644877
## resident 0.8078837 0.39882941 1.6364792
## diarrhea 1.1701536 0.83104683 1.6476322
## vomit    2.2415012 1.72392614 2.9144681
## age      1.0086575 0.99784280 1.0195894
```

```
# Third, running the mixed model including sex and age
mv_sex_age <- rma.mv(yi=logR, V=var.R + 0.01, W=wei, struct="UN", 
                mods= ~ resident + diarrhea + vomit + sex + age, method="REML", random = ~ 1 | outbreak.num)
```

```
## Warning in rma.mv(yi = logR, V = var.R + 0.01, W = wei, struct = "UN", mods
## = ~resident + : Rows with NAs omitted from model fitting.
```

```
mv_sex_age
```

```
## 
## Multivariate Meta-Analysis Model (k = 113; method: REML)
## 
## Variance Components: 
## 
##             estim    sqrt  nlvls  fixed        factor
## sigma^2    0.0895  0.2991      5     no  outbreak.num
## 
## Test for Residual Heterogeneity: 
## QE(df = 107) = 2037.8961, p-val < .0001
## 
## Test of Moderators (coefficient(s) 2:6): 
## QM(df = 5) = 26.7181, p-val < .0001
## 
## Model Results:
## 
##           estimate      se     zval    pval    ci.lb    ci.ub     
## intrcpt    -1.4912  0.4928  -3.0263  0.0025  -2.4570  -0.5254   **
## resident   -0.4074  0.4959  -0.8216  0.4113  -1.3794   0.5645     
## diarrhea    0.0875  0.1793   0.4882  0.6254  -0.2638   0.4389     
## vomit       0.6426  0.1714   3.7488  0.0002   0.3066   0.9785  ***
## sex        -0.2304  0.1144  -2.0145  0.0440  -0.4546  -0.0062    *
## age         0.0126  0.0067   1.8872  0.0591  -0.0005   0.0257    .
## 
## ---
## Signif. codes:  0 '***' 0.001 '**' 0.01 '*' 0.05 '.' 0.1 ' ' 1
```

```
mv_sex_age_exp <- cbind(exp(mv_sex_age$beta), exp(mv_sex_age$ci.lb), exp(mv_sex_age$ci.ub))
mv_sex_age_exp
```

```
##               [,1]       [,2]      [,3]
## intrcpt  0.2250957 0.08568974 0.5912966
## resident 0.6653487 0.25173851 1.7585268
## diarrhea 1.0914636 0.76809529 1.5509701
## vomit    1.9013948 1.35883794 2.6605836
## sex      0.7942203 0.63473047 0.9937854
## age      1.0126666 0.99951511 1.0259911
```

```
# Fourth, examine assoications between sex and age and the outcome and exposures

# Association between sex and logR
mixed.sex <- lmer(logR ~ sex + (1|outbreak.num))
summary(mixed.sex)
```

```
## Linear mixed model fit by REML ['lmerMod']
## Formula: logR ~ sex + (1 | outbreak.num)
## 
## REML criterion at convergence: 430.1
## 
## Scaled residuals: 
##     Min      1Q  Median      3Q     Max 
## -2.3346 -0.9605 -0.1587  0.6172  2.7443 
## 
## Random effects:
##  Groups       Name        Variance Std.Dev.
##  outbreak.num (Intercept) 0.0468   0.2163  
##  Residual                 1.0503   1.0248  
## Number of obs: 147, groups:  outbreak.num, 5
## 
## Fixed effects:
##             Estimate Std. Error t value
## (Intercept) -0.40182    0.19343  -2.077
## sex         -0.08697    0.19221  -0.452
## 
## Correlation of Fixed Effects:
##     (Intr)
## sex -0.733
```

```
confint(mixed.sex)
```

```
## Computing profile confidence intervals ...
```

```
##                  2.5 %      97.5 %
## .sig01       0.0000000  0.53376836
## .sigma       0.9132140  1.15278587
## (Intercept) -0.7748546 -0.01632663
## sex         -0.4603387  0.29777021
```

```
# Association between age and logR
mixed.age <- lmer(logR ~ age + (1|outbreak.num))
summary(mixed.age)
```

```
## Linear mixed model fit by REML ['lmerMod']
## Formula: logR ~ age + (1 | outbreak.num)
## 
## REML criterion at convergence: 457.6
## 
## Scaled residuals: 
##     Min      1Q  Median      3Q     Max 
## -2.3400 -0.7519 -0.1758  0.5046  2.4703 
## 
## Random effects:
##  Groups       Name        Variance Std.Dev.
##  outbreak.num (Intercept) 0.006629 0.08142 
##  Residual                 1.208106 1.09914 
## Number of obs: 148, groups:  outbreak.num, 6
## 
## Fixed effects:
##              Estimate Std. Error t value
## (Intercept) -0.698154   0.618036  -1.130
## age          0.002169   0.007304   0.297
## 
## Correlation of Fixed Effects:
##     (Intr)
## age -0.988
```

```
confint(mixed.age)
```

```
## Computing profile confidence intervals ...
```

```
## Warning in nextpar(mat, cc, i, delta, lowcut, upcut): Last two rows have
## identical or NA .zeta values: using minstep
```

```
## Warning in FUN(X[[i]], ...): non-monotonic profile for .sig01
```

```
## Warning in optwrap(optimizer, par = start, fn = function(x)
## dd(mkpar(npar1, : convergence code 3 from bobyqa: bobyqa -- a trust region
## step failed to reduce q
```

```
## Warning in confint.thpr(pp, level = level, zeta = zeta): bad spline fit
## for .sig01: falling back to linear interpolation
```

```
##                   2.5 %     97.5 %
## .sig01       0.00000000 0.36553551
## .sigma       0.98040076 1.23167641
## (Intercept) -1.90674279 0.50843353
## age         -0.01214825 0.01641825
```

```
# Association between sex and vomit
sex.vomit <- lm(vomit ~ sex)
summary(sex.vomit)
```

```
## 
## Call:
## lm(formula = vomit ~ sex)
## 
## Residuals:
##     Min      1Q  Median      3Q     Max 
## -0.8461  0.1538  0.2710  0.2710  0.2710 
## 
## Coefficients:
##             Estimate Std. Error t value Pr(>|t|)    
## (Intercept)  0.84615    0.06832  12.384   <2e-16 ***
## sex         -0.11718    0.07981  -1.468    0.144    
## ---
## Signif. codes:  0 '***' 0.001 '**' 0.01 '*' 0.05 '.' 0.1 ' ' 1
## 
## Residual standard error: 0.4267 on 144 degrees of freedom
##   (51 observations deleted due to missingness)
## Multiple R-squared:  0.01475,    Adjusted R-squared:  0.007908 
## F-statistic: 2.156 on 1 and 144 DF,  p-value: 0.1442
```

```
# Association between sex and diarrhea
sex.diarrhea <- lm(diarrhea ~ sex)
summary(sex.diarrhea)
```

```
## 
## Call:
## lm(formula = diarrhea ~ sex)
## 
## Residuals:
##     Min      1Q  Median      3Q     Max 
## -0.7924  0.2076  0.2076  0.2076  0.2308 
## 
## Coefficients:
##             Estimate Std. Error t value Pr(>|t|)    
## (Intercept)  0.76923    0.06609   11.64   <2e-16 ***
## sex          0.02322    0.07729    0.30    0.764    
## ---
## Signif. codes:  0 '***' 0.001 '**' 0.01 '*' 0.05 '.' 0.1 ' ' 1
## 
## Residual standard error: 0.4127 on 143 degrees of freedom
##   (52 observations deleted due to missingness)
## Multiple R-squared:  0.0006308,  Adjusted R-squared:  -0.006358 
## F-statistic: 0.09026 on 1 and 143 DF,  p-value: 0.7643
```

```
# Association between sex and resident
sex.resident <- lm(resident ~ sex)
summary(sex.resident)
```

```
## 
## Call:
## lm(formula = resident ~ sex)
## 
## Residuals:
##     Min      1Q  Median      3Q     Max 
## -0.8718  0.1282  0.2037  0.2037  0.2037 
## 
## Coefficients:
##             Estimate Std. Error t value Pr(>|t|)    
## (Intercept)  0.87179    0.06220   14.02   <2e-16 ***
## sex         -0.07550    0.07257   -1.04      0.3    
## ---
## Signif. codes:  0 '***' 0.001 '**' 0.01 '*' 0.05 '.' 0.1 ' ' 1
## 
## Residual standard error: 0.3884 on 145 degrees of freedom
##   (50 observations deleted due to missingness)
## Multiple R-squared:  0.00741,    Adjusted R-squared:  0.0005646 
## F-statistic: 1.082 on 1 and 145 DF,  p-value: 0.2999
```

```
# Association between age and vomit
age.vomit <- lm(vomit ~ age)
summary(age.vomit)
```

```
## 
## Call:
## lm(formula = vomit ~ age)
## 
## Residuals:
##     Min      1Q  Median      3Q     Max 
## -0.7974 -0.6896  0.2908  0.3034  0.3363 
## 
## Coefficients:
##              Estimate Std. Error t value Pr(>|t|)    
## (Intercept)  0.888250   0.256049   3.469 0.000688 ***
## age         -0.002163   0.003030  -0.714 0.476517    
## ---
## Signif. codes:  0 '***' 0.001 '**' 0.01 '*' 0.05 '.' 0.1 ' ' 1
## 
## Residual standard error: 0.4572 on 145 degrees of freedom
##   (50 observations deleted due to missingness)
## Multiple R-squared:  0.003501,   Adjusted R-squared:  -0.003371 
## F-statistic: 0.5095 on 1 and 145 DF,  p-value: 0.4765
```

```
# Association between age and diarrhea
age.diarrhea <- lm(diarrhea ~ age)
summary(age.diarrhea)
```

```
## 
## Call:
## lm(formula = diarrhea ~ age)
## 
## Residuals:
##     Min      1Q  Median      3Q     Max 
## -0.8628  0.1570  0.1760  0.1892  0.3421 
## 
## Coefficients:
##             Estimate Std. Error t value Pr(>|t|)  
## (Intercept) 0.553917   0.220362   2.514    0.013 *
## age         0.003059   0.002608   1.173    0.243  
## ---
## Signif. codes:  0 '***' 0.001 '**' 0.01 '*' 0.05 '.' 0.1 ' ' 1
## 
## Residual standard error: 0.3935 on 145 degrees of freedom
##   (50 observations deleted due to missingness)
## Multiple R-squared:  0.009396,   Adjusted R-squared:  0.002564 
## F-statistic: 1.375 on 1 and 145 DF,  p-value: 0.2428
```

```
# Association between age and resident
age.resident <- lm(resident ~ age)
summary(age.resident)
```

```
## 
## Call:
## lm(formula = resident ~ age)
## 
## Residuals:
##      Min       1Q   Median       3Q      Max 
## -0.57821 -0.04117  0.00737  0.05148  0.54020 
## 
## Coefficients:
##             Estimate Std. Error t value Pr(>|t|)    
## (Intercept) -0.03750    0.08622  -0.435    0.664    
## age          0.01184    0.00102  11.611   <2e-16 ***
## ---
## Signif. codes:  0 '***' 0.001 '**' 0.01 '*' 0.05 '.' 0.1 ' ' 1
## 
## Residual standard error: 0.1541 on 146 degrees of freedom
##   (49 observations deleted due to missingness)
## Multiple R-squared:  0.4801, Adjusted R-squared:  0.4765 
## F-statistic: 134.8 on 1 and 146 DF,  p-value: < 2.2e-16
```

3. Including cases with REi = 0

To include cases with REi = 0, use the original dataset (before excluding cases with REi=0) and add 0.01 to REi estimates. Then take the log of this new REi variabe and run the model as before.

```
outbreaks$R_SA <- outbreaks$R + 0.01
outbreaks$logR_SA <- log(outbreaks$R_SA)

meta_SA <- outbreaks[!is.na(outbreaks$Vomit),]
meta_SA <- meta_SA[!is.na(meta_SA$Diarrhea),]
meta_SA <- meta_SA[!is.na(meta_SA$Resident),]

logR_SA <- meta_SA$logR_SA
resident_SA <- meta_SA$Resident
diarrhea_SA <-meta_SA$Diarrhea
vomit_SA <- meta_SA$Vomit
outbreak.num_SA <- factor(meta_SA$Outbreak_Number)
var.R_SA <- meta_SA$var_R
wei_SA <- meta_SA$weight

mv_SA <- rma.mv(yi=logR_SA, V=var.R_SA + 0.01, W=wei_SA, struct="UN",
                mods= ~ resident_SA + diarrhea_SA + vomit_SA, method="REML", 
                random = ~ 1 | outbreak.num_SA)
mv_SA
```

```
## 
## Multivariate Meta-Analysis Model (k = 204; method: REML)
## 
## Variance Components: 
## 
##             estim    sqrt  nlvls  fixed           factor
## sigma^2    0.8859  0.9412      6     no  outbreak.num_SA
## 
## Test for Residual Heterogeneity: 
## QE(df = 200) = 12745.8168, p-val < .0001
## 
## Test of Moderators (coefficient(s) 2:4): 
## QM(df = 3) = 1643.9327, p-val < .0001
## 
## Model Results:
## 
##              estimate      se     zval    pval    ci.lb    ci.ub     
## intrcpt       -3.7549  0.3887  -9.6598  <.0001  -4.5168  -2.9930  ***
## resident_SA    1.1857  0.0360  32.9117  <.0001   1.1151   1.2563  ***
## diarrhea_SA    0.5909  0.0336  17.5854  <.0001   0.5250   0.6567  ***
## vomit_SA       0.9390  0.0332  28.2460  <.0001   0.8738   1.0041  ***
## 
## ---
## Signif. codes:  0 '***' 0.001 '**' 0.01 '*' 0.05 '.' 0.1 ' ' 1
```

```
mv_SA_exp <- cbind(exp(mv_SA$beta), exp(mv_SA$ci.lb), exp(mv_SA$ci.ub), mv_SA$pval)
mv_SA_exp
```

```
##                   [,1]       [,2]       [,3]          [,4]
## intrcpt     0.02340251 0.01092416 0.05013454  4.466780e-22
## resident_SA 3.27298617 3.04984712 3.51245096 1.497326e-237
## diarrhea_SA 1.80552499 1.69045670 1.92842591  3.185678e-69
## vomit_SA    2.55732313 2.39601610 2.72948983 1.594091e-175
```
