# Supplementary material for: Quantifying the roles of vomiting, diarrhea, and residents vs. staff in norovirus transmission in U.S. nursing home outbreaks
Source: PLoS Comput Biol. 2020 Mar 25;16(3):e1007271. doi: 10.1371/journal.pcbi.1007271 (PMC7135310; doi:10.1371/journal.pcbi.1007271)
Supplement: S1 Poster — This poster shows preliminary results from the paper and was presented in March 2018 at the NoroCORE Final Showcase Meeting in Atlanta, GA, USA. (PDF) [file pcbi.1007271.s005.pdf]

Norovirus infectiousness in long-term care facility outbreaks: estimating who infected whom from epidemic curves

Carly L Adams[1], Benjamin A Lopman[1]

Background

- Over half of all norovirus outbreaks reported in the US, and 90% of those found to be non-foodborne, occur in long-term care facilities (LTCFs). [2]
- Older adults and residents in LTCFs may be at heightened risk due to increased risk of exposure and subsequent illness and/or heightened risk of severe outcome. [3]

Objectives

- Estimate the effective reproduction number (R_E)* of norovirus LTCF outbreaks and examine changes over the course of an outbreak. [4]
- Estimate case-specific R_E's and examine differences by case characteristics and symptoms.

Data

- De-identified line lists from six separate outbreaks were provided by the South Carolina Department of Health and Environmental Control.
- Outbreaks occurred during the 2014/15 and 2015/16 norovirus seasons and ranged in size from 12-52 cases.
- A total of 209 cases (157 residents and 49 staff members) were affected in these outbreaks.
- Of all cases, 55% were female and 20% were male.
- Seventy-eight percent of cases reported diarrhea and 74% reported vomiting.

**Defined as the average number of secondary cases that one index case generates over the course of its infectious* period.

Methods

- Estimated R_E using a likelihood-based estimation procedure, as previously developed and described by Wallinga and Teunis, to infer the temporal pattern of R_E from observed LTCF norovirus outbreak curves. [5, 6]
- Only onset dates of cases and knowledge of the serial interval of norovirus (the duration between the dates of symptom onset) were required to estimate R_E.

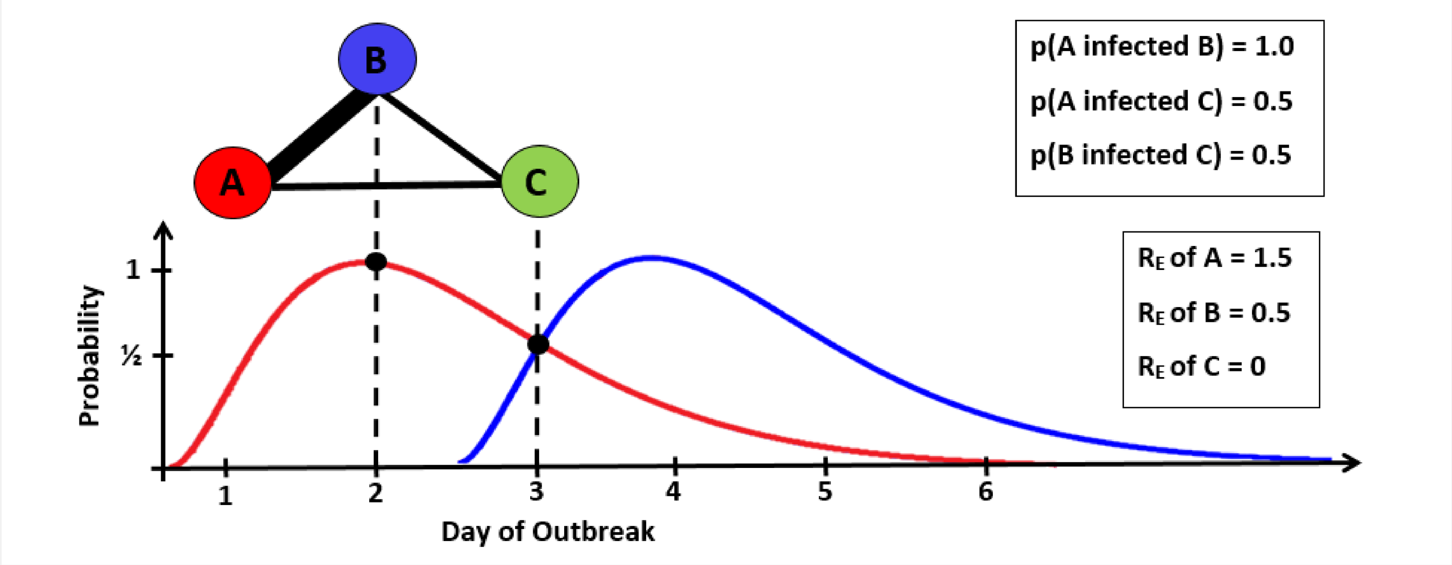

The figure above illustrates the Wallinga-Teunis method. A transmission tree for a hypothetical outbreak of three cases, with gamma probability distributions for cases A (red) and B (blue), is shown. Widths of edges correspond to probability of transmission and probabilities sum to R_E.

Conclusions

- Residents appear to have a greater average R_E compared to staff members.
- Male residents appear to have a greater average R_E compared to female residents.
- Estimates suggest vomiting plays a key role in norovirus transmission in LTCFs.
- Estimates suggest age plays a role in norovirus transmission in LTCFs.

Acknowledgements: We sincerely thank David Young [7], Paul Gastanaduy [8], Prabasaj Paul [8], Aron Hall [8] and Zachary Marsh [8] for their active support of this evaluation.

R_E by Case Characteristics

Case Characteristics	Average R_E (95% CI)
Case Status	
Resident	1.03 (0.79, 1.28)
Staff	0.75 (0.40, 1.11)
Sex - Staff	
Male	0.45 (0.18, 0.73)
Female	1.15 (0.48, 1.81)
Sex - Residents	
Male	1.30 (0.61, 1.99)
Female	0.96 (0.69, 1.24)
Age Range (years) - Residents*	
< 70	0.84 (0.27, 1.41)
70 – 79	0.96 (0.23, 1.70)
80 – 89	1.07 (0.71, 1.42)
≥ 90	1.24 (0.70, 1.79)

**All staff were < 70 years old.*

R_E by Reported Symptoms

Reported Symptom(s)	Average R_E (95% CI)
Fever	
No	0.96 (0.71, 1.21)
Yes	0.86 (0.51, 1.20)
Diarrhea	
No	0.84 (0.42, 1.25)
Yes	0.99 (0.75, 1.22)
Vomiting	
No	0.54 (0.29, 0.78)
Yes	1.11 (0.85, 1.37)
Diarrhea & Vomiting	
No	0.69 (0.46, 0.92)
Yes	1.19 (0.87, 1.51)

Norovirus Outbreak Epidemic Curves and Effective Reproduction Ratios*

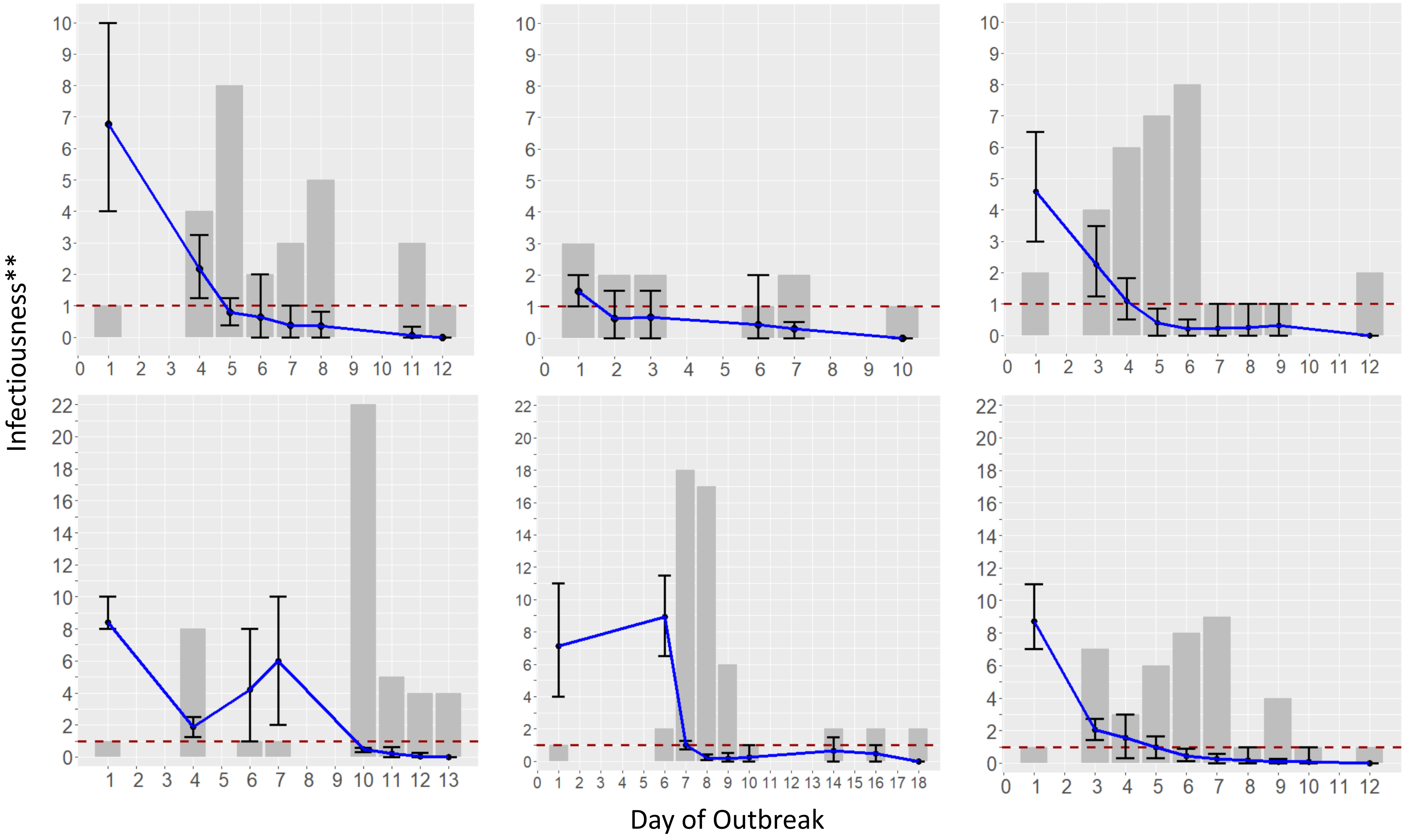

**Case counts (gray bars) and R_E's (point estimates with corresponding 95% confidence intervals) for six separate LTCF norovirus outbreaks. **Note the change in scale.*

1. Department of Epidemiology, Rollins School of Public Health, Emory University
2. "U.S. Trends and Outbreaks." 24 June 2016. Centers for Disease Control and Prevention. https://www.cdc.gov/norovirus/trends-outbreaks.html.
3. Lindsay, Lisa, et al. "A decade of norovirus disease risk among older adults in upper-middle and high income countries." *BMC Infectious Diseases.* 2015.
4. Cowlng, Benjamin, et al. "The effective reproduction number of pandemic influenza: prospective estimation". *Epidemiology*. 2010 Nov; 21(6): 842–846.
5. Wallinga, Jaco & Teunis, Peter. "Different epidemic curves for SARS reveal similar impacts of control measures." *Am J Epidemiol.* 2004.
6. Heijne, Janneke C.M., et al. "Quantifying transmission of norovirus during an outbreak." *Epidemiology*, Vol 23, No. 2, 2012 March.
7. South Carolina Department of Health and Environmental Control
8. Centers for Disease Control and Prevention, Office of Infectious Diseases, National Center for Immunization and Respiratory Diseases
