# Supplementary material for: Quantifying the roles of vomiting, diarrhea, and residents vs. staff in norovirus transmission in U.S. nursing home outbreaks
Source: PLoS Comput Biol. 2020 Mar 25;16(3):e1007271. doi: 10.1371/journal.pcbi.1007271 (PMC7135310; doi:10.1371/journal.pcbi.1007271)
Supplement: Attachment — Submitted filename: Initial review.docx [file pcbi.1007271.s006.docx]

In this clearly written manuscript, Adams et al. explore risk factors for the transmission of norovirus during nursing home outbreaks. To accomplish this goal, they analyze line listing data from six nursing homes in South Carolina. They first use the Wallinga-Teunis approach to determine a reproduction number for each norovirus case and then perform a regression on the reproduction numbers. The result of their regression show that vomiting, diarrhea, and index infections are all associated with higher transmission. The affect is most pronounced for residents who vomit.

By focusing on traits that affect transmissibility of norovirus, as compared to the traditional approach of identifying the risk factors for susceptibility of acquiring disease, the manuscript provides useful perspective on norovirus transmission dynamics and control strategies. Although the finding that diarrhea and vomiting impact transmission is not surprising, the ability to provide quantitative characterization of this effect is novel. The methods appear reasonable, but some clarification is needed concerning the estimate of confidence intervals, the interpretation of the coefficients of the regression and whether the manuscript’s claim that residents transmit more than staff reaches statistical significance.

Major concerns

- Please explain how confidence intervals for regression coefficients are determined – particularly given there are uncertainties related to the Wallinga-Teunis inference as well as the regression coefficients themselves.
- Please comment on whether regression on individual reproduction numbers obtained via Wallinga-Teunis estimation has been validated. If there are no good references to support this novel methodology, then some confirmatory method is needed to confirm confidence interval estimation (such as a parametric bootstrap based on simulated data).
- Please clarify the interpretation of the regression coefficients. For example in lines 269-272 the wording makes it appear that vomiting causes an additional 2.74 infections, but the equation on line 180 indicates it is a multiplicative factor.
- Lines 258-260**.** I am confused by the inclusion of results with p-values of 0.11 and 0.24. More justification is needed to support the manuscript’s claim that residents transmit more than staff.

Minor concerns:

- Figure 2 is hard to interpret. In understand the point is to show that folks who vomit have higher R, but instead it emphasizes that most cases have R between 0 and 1. I’d reconsider how to present this data (e.g. consider normalizing each bar to 100%)
- Line 253. Please clarify why the p-value is so small even when there is significant overlap of the IQR ranges.
- Line 257. Did you try including an interaction term for vomiting and diarrhea (since they frequently co-exist)? If not, please explain why.

Suggested edits:

- Line 145 – my understanding of the Wallinga-Teunis approach is to weight the probability of a transmission link based on the probability density for the serial interval (as compared to selecting the link associated with highest probability as suggested by the manuscript’s wording). Can you clarify?
- Line 156 – Please clarify the number used for C. Is it the total number of residents and staff in the nursing home?
- Line 190 – Perhaps provide a quick explanation for what the Kruskal-Wallis test is
- Line 213 – Do you know if any visitors were involved (e.g. cases) in the outbreak?
- Line 221 – For Table 1, Do you have information on the overall population of the nursing home (e.g. # residents, # staff, average age, gender ratio?). If so I’d include it.
- Line 238 – In describing the significance of Figure 1, can you explain what outbreak control interventions were implemented and the timing of when interventions were begun?
- Line 308 – I’d argue that the combination of Wallinga-Teunis and linear regression requires quite a number of assumptions (even though it is a reasonable approach). Long and short, I think it is best to just say what the assumptions are.
- Line 337-340 I don’t understand what is being said here. And I don’t understand the link to Ref 27. Can you clarify?
- Line 350-352 I think the mobility of staff vs residents actually argues against this line of thought. Rather, I wonder if staff’s greater independence allows them to recognize symptoms earlier and self-quarantine themselves?
- Lines 386-388 Presumably these infection control measures are already implemented? Any chance you can be more specific about improvements that can be made?
