# Supplementary material for: Quantifying the roles of vomiting, diarrhea, and residents vs. staff in norovirus transmission in U.S. nursing home outbreaks
Source: PLoS Comput Biol. 2020 Mar 25;16(3):e1007271. doi: 10.1371/journal.pcbi.1007271 (PMC7135310; doi:10.1371/journal.pcbi.1007271)
Supplement: Attachment — Submitted filename: Response to Reviewers.docx [file pcbi.1007271.s007.docx]

Norovirus Transmission in Nursing Homes manuscript

PLOS Computational Biology Revisions

Updated: Dec 5, 2019

**Requests from the reviewers:**

Reviewer #1:

Major concerns

- Please explain how confidence intervals for regression coefficients are determined – particularly given there are uncertainties related to the Wallinga-Teunis inference as well as the regression coefficients themselves.

Response: This is an important point to clarify and we thank the reviewer for raising this question. Uncertainty from the *R_Ei_* estimates were not originally included in the confidence intervals for the regression coefficients. To address this, we took a meta-regression approach and weighted the outcomes, with weights equal to the inverse of the sum of the three variances for each *R_Ei_* estimate (see below for explanation). We found that results changed only slightly and conclusions regarding the relative importance of vomiting did not change.

Edits to text (lines 149-151): “… and corresponding 95% confidence interval … and confidence interval.”

Edits to text (lines 179-184): “Furthermore, because each *R_Ei_* estimate had its own uncertainty, we used a meta-analysis approach and incorporated *R_Ei_* uncertainties by using inverse variance weighting. Weights were equal to the inverse of the sum of the three variance components: 1. variance from estimation of REi (unique to each onset date), 2. within-outbreak variance (unique to each outbreak), and 3. between-outbreak variance (equal for all estimates).”

Edits to text (lines 295-298): “In the final multivariable model, cases who vomited infected 2.12 (95% CI: 1.68, 2.68) times the number of individuals as non-vomiters, cases with diarrhea infected 1.39 (95% CI: 1.03, 1.87) times the number of individuals as cases without diarrhea, and resident-cases infected 1.53 (95% CI: 1.15, 2.02) times the number of individuals as staff-cases (Fig 3).”

Figure 3: We updated results (coefficient estimates and 95% CIs) after incorporating weights into the mixed linear regression analyses.

Edits to text (331-332): “…with a log-transformed outcome variable (*R_Ei_*), inverse-variance weighting, …”

Edits to supporting information (S1 RMarkdown File “Estimating Individual Reproduction Numbers”): Included instructions to copy variances of *R_Ei_* estimates into the excel file.

Edits to supporting information (S3 RMarkdown file “Serial Interval Sensitivity Analysis”): Changed the code so that variances for *R_Ei_* estimates are also included in outputs.

Edits to supporting information (S2 RMarkdown File “Regression Analyses”): We added this file to the supporting information. This file includes information on how to perform the multivariate mixed linear regression model with weighted *R_Ei_* estimates. It also includes additional regression analyses that we performed: examining the infectiousness of index cases and associations between onset day and vomiting, diarrhea, and resident. It also includes data for the following sensitivity analyses: varying the serial interval lengths, adding age and/or sex to the model, and including cases with *R_Ei_*=0 in the regression analysis.

Edits to supporting information (S1 File “Outbreak Information”): Added columns for variances of *R_Ei_* estimates, a column for sex, and a column for age.

Edits to supporting information: Removed SAS file "Mixed linear regression analyses" as all updated analyses used R.

- Please comment on whether regression on individual reproduction numbers obtained via Wallinga-Teunis estimation has been validated. If there are no good references to support this novel methodology, then some confirmatory method is needed to confirm confidence interval estimation (such as a parametric bootstrap based on simulated data).

Response: The Wallinga-Teunis method itself has been used been used several times before to infer reproduction numbers from epidemic curves. While our approach to subsequently analyze the outputs may not have been formally validated, we use a standard multivariate mixed linear regression to examine the associations between reproduction numbers and case characteristics. We agree that reproduction numbers are different than typical outcome variables because they have their own uncertainty, which we did not originally take into account when calculating confidence intervals for the regression estimates. To address this, we changed our regression analyses so that reproduction numbers were weighted by the inverse of their variances and found that conclusions remained the same.

Edits to text: See above.

- Please clarify the interpretation of the regression coefficients. For example in lines 269-272 the wording makes it appear that vomiting causes an additional 2.74 infections, but the equation on line 180 indicates it is a multiplicative factor.

Response: We thank the reviewer for bringing this to our attention and apologize for the misinterpretation. We have made corrections to interpretations of exponentiated regression coefficients throughout the text.

Edits to text (lines 47-51): “Cases who vomited infected 2.12 (95% CI: 1.68, 2.68) times the number of individuals as non-vomiters, cases with diarrhea infected 1.39 (95% CI: 1.03, 1.87) times the number of individuals as cases without diarrhea, and resident-cases infected 1.53 (95% CI: 1.15, 2.02) times the number of individuals as staff-cases.”

Edits to text (lines 295-298): “…cases who vomited infected 2.12 (95% CI: 1.68, 2.68) times the number of individuals as non-vomiters, cases with diarrhea infected 1.39 (95% CI: 1.03, 1.87) times the number of individuals as cases without diarrhea, and resident-cases infected 1.53 (95% CI: 1.15, 2.02) times the number of individuals as staff-cases (Fig 3).”

Edits to text (lines 304-305): “…index cases infected 3.96 (95% CI: 2.44, 6.44) times the number of individuals as non-index cases…”

Edits to text (lines 318-319): “…cases who vomited infected 1.90 (95% CI: 1.36, 2.66) times the number of individuals as non-vomiters.

Edits to text (lines 321-323): “…cases with diarrhea infected 1.09 (95% CI: 0.77, 1.55) times the number of individuals as cases without diarrhea and resident-cases infected 0.67 (95% CI: 0.25, 1.76) times the number of individuals as staff-cases…”

- Lines 258-260**.** I am confused by the inclusion of results with p-values of 0.11 and 0.24. More justification is needed to support the manuscript’s claim that residents transmit more than staff.

Response: We thank the reviewer for raising the issue. While differences in median *R_Ei_* estimates between cases with both vomiting and diarrhea and vomiting alone and between residents and staff were not significant, with overlapping IQRs, we presented these results to show the patterns.

For vomiting and diarrhea vs. vomiting alone (p-value = 0.24): because the difference in *R_Ei_* estimates between cases with both vomiting and diarrhea and diarrhea alone was statistically significant, whereas the difference between cases with both vomiting and diarrhea and vomiting alone was not, this actually supports our claim that vomiting appears to be driving transmission in these settings.

For the difference between residents and staff (p-value = 0.11): we agree that this information alone would not be enough to support our claim that residents are more infectious than staff. However, these results taken together with the regression results, which showed that residents had significantly greater *R_Ei_* estimates compared to staff, support this claim.

Minor concerns:

- Figure 2 is hard to interpret. In understand the point is to show that folks who vomit have higher R, but instead it emphasizes that most cases have R between 0 and 1. I’d reconsider how to present this data (e.g. consider normalizing each bar to 100%)

Response: We agree with the reviewer that Fig 2 was difficult to interpret, and appreciate the suggestions.

Edits to text (Figure 2): We normalized each bar to 100% and increased the width of the black boxes to emphasize that index cases have higher *R_Ei_* values and tend to be residents who vomited.

Edits to text (lines 268-270): “Percentage of cases by individual reproduction number^a^, REi, for the following categories: residents who vomited, staff who vomited, and residents/staff who did not vomit; index cases are outlined in black.”

- Line 253. Please clarify why the p-value is so small even when there is significant overlap of the IQR ranges.

Response: Because we used the Kruskal-Wallis test, which is a non-parametric test that pools the data (across groups) and ranks observations from lowest to highest, it is possible to have statistically significant results even if IQRs overlap. For the difference between median *R_Ei_* estimates for cases with diarrhea only compared to cases with vomiting and diarrhea, the Kruskal-Wallis chi-squared test statistic was 9.7 with 1 degree of freedom, giving us a p-value of 0.002. This means that significantly more *R_Ei_* values for cases with vomiting and diarrhea were ranked above those with diarrhea only, compared to the number of *R_Ei_* values for cases with diarrhea only that were ranked above cases with diarrhea and vomiting. We added text to clarify this.

Edits to text (lines 206-208): “The Kruskal-Wallis test pools data across groups and ranks observations from lowest to highest, making it possible for results to be statistically significant even when IQRs overlap”

- Line 257. Did you try including an interaction term for vomiting and diarrhea (since they frequently co-exist)? If not, please explain why.

Response: Yes, we did try including an interaction term for vomiting and diarrhea. However, due to the small sample size (number of cases with: diarrhea only = 51; vomiting only = 41; diarrhea and vomiting = 113) and problems with collinearity, we decided to exclude the interaction term from the model. When an interaction term was included in the model, the condition index (CI) increased to 36 and the Variance Decomposition Proportions (VDPs) for vomiting, diarrhea, and vomit*diarrhea were all well above 0.5 (0.97, 0.97, and 0.95, respectively), indicating problems of collinearity. However, the potential interaction between vomit and diarrhea is something we would like to examine in the future with a larger sample size. We added text to clarify why we did not include an interaction term in the model and indicated that this should be studied in the future.

Edits to text (lines 187-190): “We also considered including an interaction between diarrhea and vomit in the model, however we excluded this interaction term from the final model due to issues with collinearity with the individual vomit and diarrhea coefficients.”

Edits to text (lines 446-447): “Future studies should also further examine the association between case infectiousness and the interaction between vomiting and diarrhea.”

Suggested edits:

- Line 145 – my understanding of the Wallinga-Teunis approach is to weight the probability of a transmission link based on the probability density for the serial interval (as compared to selecting the link associated with highest probability as suggested by the manuscript’s wording). Can you clarify?

Response: We agree that this wording should be changed and appreciate the suggestion.

Edits to text (lines 140-141): Changed wording to “probability distribution” and specified that the serial interval is for a specific infectious disease.

Edits to text (lines 145-149): “Briefly, this method uses the difference in symptom onsets dates between cases and the probability distribution of the serial interval to calculate the relative likelihood that cases with earlier symptom onset dates infected cases with later symptom onset dates. The relative likelihoods are then summed to estimate the *R_Ei_* for each case.”

- Line 156 – Please clarify the number used for C. Is it the total number of residents and staff in the nursing home?

Response: The number used for C is the number of cases in the outbreak (minus the number of cases with illness onset on day 1). We have clarified this in the text and appreciate the question. Furthermore, we changed the notation to indicate that calculations excluded index cases.

Edits to text (line 157): $p_{i}= \frac{1-\sum_{1}^{i} C_{i}}{C}$

Edits to text (lines 158-159): “…or the total number of cases in the outbreak minus the number of index cases…”

Edits to text (line 159): $\sum_{1}^{i} C_{i}$

- Line 190 – Perhaps provide a quick explanation for what the Kruskal-Wallis test is

Response: We agree that this would be useful information to include and thank the reviewer for this suggestion. We have added a brief description to the text.

Edits to text (lines 204-206): “… using the Kruskal-Wallis test, a nonparametric test used to assess differences of a continuous dependent variable by a categorical independent variable when the assumptions of one-way ANOVA are not met.”

- Line 213 – Do you know if any visitors were involved (e.g. cases) in the outbreak?

Response: To our knowledge, only nursing home residents and staff were involved in the outbreaks. We’ve added text to specify that no visitors were involved.

Edit to text (lines 228-229): “We have no data indicating that visitors were involved in the outbreaks.”

- Line 221 – For Table 1, Do you have information on the overall population of the nursing home (e.g. # residents, # staff, average age, gender ratio?). If so I’d include it.

Response: We agree that this information would be beneficial to include, but did not have access to this information. We plan to collect these data for future studies.

- Line 238 – In describing the significance of Figure 1, can you explain what outbreak control interventions were implemented and the timing of when interventions were begun?

Response: We agree that it’s important to examine the impact of control measures on transmission dynamics. However control measure information (whether and which control measures were implemented, when they were implemented, for how long, etc.) were not available for this study.

- Line 308 – I’d argue that the combination of Wallinga-Teunis and linear regression requires quite a number of assumptions (even though it is a reasonable approach). Long and short, I think it is best to just say what the assumptions are.

Response: We agree with the reviewer and appreciate the suggestion. We added to the text to include a list of assumptions that were made (and deleted later text that listed some of these assumptions so as not to be redundant).

Edits to text: We removed the following text: “The novel application of our modeling methods to estimate reproduction numbers required few assumptions regarding norovirus transmission.”

Edits to text (lines 344-347): “We assumed the following: transmission of infection occurred only among reported cases, asymptomatic cases did not play a role in transmission, all reported cases were part of the same outbreak, and the serial interval for norovirus in this setting is gamma distributed with a mean of 3.6 days.”

- Line 337-340 I don’t understand what is being said here. And I don’t understand the link to Ref 27. Can you clarify?

Response: Here we are saying that while the median *R_Ei_* may be approximately 1 (as we saw in our outbreaks), index cases may be hyperinfectious (i.e., superspreaders), meaning that these hyperinfectious index cases (who may be hyperinfectious due to vomiting, etc.) are required for an outbreak to be initiated. We agree that the reference to the paper on superspreaders was not relevant and removed it. We added a reference to a paper that found that norovirus hospital outbreaks were associated with index cases who vomited.

Edits to text: Removed reference 27.

Edits to text (lines 384-385): “This hypothesis is supported by a recent paper that found that vomiting in norovirus index cases was associated with an increased risk of nosocomial outbreaks [28].”

- Line 350-352 I think the mobility of staff vs residents actually argues against this line of thought. Rather, I wonder if staff’s greater independence allows them to recognize symptoms earlier and self-quarantine themselves?

Response: Because we did not have information on residents’ levels of dependency, we were unable to examine the association between levels of dependency and transmission. We hypothesized that more mobile residents would be moving around to communal areas where they would be more likely to infect others, but we agree that it is also possible that more independent residents would self-quarantine, thus limiting spread to staff and other residents. We thank the reviewer for making this suggestion.

Edits to text (lines 397-399): “Conversely, more self-sufficient residents may be more likely to identify symptoms of norovirus and self-quarantine, thus transmitting less.”

- Lines 386-388 Presumably these infection control measures are already implemented? Any chance you can be more specific about improvements that can be made?

Response: Presumably these infection control measures were implemented, however we lack data on what was implemented and when. Furthermore, antiemetic drugs are not widely recommended for use in controlling norovirus outbreaks in healthcare settings. We added text to specify that this is an area where improvements could be made.

Edits to text (lines 438-443): “While cleaning with a chlorine-based disinfectant and isolation of cases are currently recommended control strategies, there are generally little data about the implementation and timing of these control measures in norovirus outbreaks [10]. Furthermore, the use of antiemetic drugs for the prevention and control of norovirus outbreaks is not widely recommended [31, 32]. The important role of vomiting in transmission shown in our results suggests that the use of antiemetic drugs in nursing home outbreaks should be further considered.”

Reviewer #2:

Line 111: As the outbreaks come from two seasons- is there any evidence for different transmission dynamics between the two?

Response: We thank the reviewer for raising this question and agree that this would be useful information. We have added text to address this question.

Edits to text (lines 244-250): “Of the six outbreaks, four occurred during the 2014-2015 norovirus season (total cases: 141) and two occurred during the 2015-2016 norovirus season (total cases: 68). Due to the limited number of outbreaks, we were unable to make formal comparisons. A greater proportion of cases reported diarrhea, vomiting, and were nursing home residents in the 2014-2015 season (78%, 81%, and 83%, respectively) compared to the 2015-2016 season (68%, 66%, and 72%, respectively). Average *R_Ei_* estimates were similar between the two seasons (2014-2015: 0.95; 2015-2016: 0.97).”

Line 131: Estimates of Heijne et al. go up to R0 ~14.

Response: We thank the reviewer for bringing this to our attention. The Heijne et al. estimate of 14.05 was for the effective reproduction number for norovirus. We’ve added text to clarify this.

Edits to text (lines 133-134): “Estimates for the *R_0_* and *R_E_* of norovirus vary widely (1.1 to 7.2 and 0.85 to 14.05, respectively), and depend on differences in settings [18].”

Line 150: It is worth noting that index cases, due to their limited nature (ie. there will be only 1-3 per outbreak) will have the most uncertain Rei. In this way, including further outbreaks in this analysis may produce a better estimate of how infectious index cases are compared to other cases (not a suggestion for this paper).

Response: Indeed, we agree that it would be beneficial to examine index cases further using a larger dataset. We plan to do so for future studies.

Line 154: Stating that the entire population is susceptible is a strong assumption, as mentioned in the discussion. It is difficult to establish pre-existing immunity; I would suggest a sensitivity analysis exploring the possibility that eg. 90% of the nursing home population is susceptible at the start.

Response: We agree that this is an important point. Because assuming 100% of the population is susceptible is the most extreme assumption about susceptibility, decreasing the proportion susceptible only increases the differences in *R_Ei_* estimates seen between index cases (onset on day 1) and *R_Ei_* estimates for cases from days 2 through 4, as seen below:

We have added text to clarify this.

Edits to text (lines 377-379): “Because this was the most extreme assumption, the differences between *R_0,1_* and *R_0,2-4_* estimates became even more pronounced when the population was assumed to have some level of immunity (e.g., 50% susceptibility).”

Line 187: The outbreaks are assumed to be independent but they all occurred in South Carolina within two years. Could you comment on the likelihood that the outbreaks may be related?

Response: Nursing home norovirus outbreaks are the observable ‘tip of the iceberg’ and likely result from introductions of norovirus from the community. While there may be distant links though the community, it is probably safe to assume that transmission within a nursing home is independent of other nursing homes. Similarly, it’s probably safe to assume that residents were not transferred between the six nursing homes in this study within the times that outbreaks took place. With more than 180 separate nursing home facilities in South Carolina, we think it’s unlikely that they’re related.

Line 190: Please add a note to explain the Kruskal-Wallis test.

Response: We agree that this would be useful information to include and thank the reviewer for this suggestion. We have added a brief description to the text.

Edits to text (lines 204-206): “… using the Kruskal-Wallis test, a nonparametric test used to assess differences of a continuous dependent variable by a categorical independent variable when the assumptions of one-way ANOVA are not met.”

Line 216: Were the excluded cases distributed evenly between different outbreaks?

Response: Each outbreak had at least one excluded case, with outbreak 3 having the most excluded cases, with 5 cases excluded. We agree that this would be useful information to include in the results and have added a column to table 1 to indicate the number and percent of cases excluded from the regression analyses for each outbreak.

Edits to text (Table 1): Added column 3.

Edits to text (line 238): “^a^Cases excluded from the regression.”

Line 222: Many of the cases were not lab confirmed for norovirus, is it safe to assume these are from the same outbreak?

Response: While there is no way to know for certain if all cases were outbreak-associated, as we list in the limitations, symptoms of all cases were consistent with norovirus gastroenteritis, as was the timing between cases. Furthermore, all outbreaks met the CDC National Outbreak Reporting System (NORS) definition of a confirmed outbreak: two or more cases of similar illness associated with a common exposure with two or more lab-confirmed cases. Given limited resources, state health departments typically only test stool specimens from 3-6 symptomatic individuals, and other cases involved in the outbreak are assumed to also be infected with norovirus. Regardless, it is possible that some included cases were not outbreak-associated, which could result in overestimates or underestimates of transmission. On the other hand, case finding is difficult in nursing homes, where gastroenteritis symptoms are common, so it’s also possible that cases were missed.

Line 228: Index cases are highly uncertain, can you comment more on the reporting of the index case ie. what is the chance that earlier cases were missed?

Response: We agree that identifying index cases is challenging, and that some ‘true’ index cases may have been missed. However, nursing homes residents are typically closely monitored for symptoms and cases promptly reported. We have added text to specify this as a limitation.

Edits to text (lines 409-410): “Relatedly, index cases may have been misclassified if true index cases were missed.”

generally: Asymptomatic infection: how would this affect the estimates?

Response: We thank the reviewer for raising this important question. Transmission from asymptomatically infected persons could result in overestimates of reproduction numbers if cases that asymptomatic individuals infected were incorrectly attributed symptomatic cases. However, this could also result in unbiased estimates if subsequent generations had the same proportion of asymptomatic infections. Regardless, this is likely a minor bias. Sukhrie et al. (“Nosocomial Transmission of Norovirus Is Mainly Caused by Symptomatic Cases”, 2012) found that the contribution of asymptomatic individuals to transmission was far lower than that of symptomatic individuals in nosocomial outbreaks, leading us to believe that bias from asymptomatic cases is likely minimal. We added text to specify this.

Edits to text (lines 410-414): “For norovirus, it’s also possible that asymptomatic cases could contribute to transmission. However, studies have found that asymptomatic individuals in nosocomial outbreaks contribute significantly less to transmission than symptomatic individuals, leading us to believe that bias from missing asymptomatic cases is likely minimal [28].”

Fig 1: The bottom left outbreak has a break between the index and secondary cases, how likely is that intermediate cases were missed?

Response: While the time between the index case and secondary cases was slightly longer in this outbreak, we still believe it’s likely that the index case (with symptoms onset on day 1) infected the secondary cases with symptom onsets on day 6. While we assumed an average serial interval of 3.6 days, norovirus can be spread for several days to weeks after illness onset. Furthermore, environmental transmission is common with norovirus, which could also have led to the longer serial interval. However, it’s also possible that there were undetected cases during that interval.

Fig 2: Generally the paper and supplementary markdown documents are presented well. As such, I think this figure could be improved. Perhaps with index cases as dots rather than boxes. Additionally, with the grey scale bars, it may be better to have a white background- something like theme_bw().

Response: We agree with the reviewer that Fig 2 could be improved, and appreciate the suggestions.

Edits to text (Figure 2): We normalized each bar to 100% and increased the width of the black boxes to emphasize that index cases have higher *R_Ei_* values and tend to be residents who vomited.

Edits to text (lines 268-270): “Percentage of cases by individual reproduction number^a^, REi, for the following categories: residents who vomited, staff who vomited, and residents/staff who did not vomit; index cases are outlined in black.”

Line 249: The conclusion that index cases are more infectious could be explained by other factors. These cases are most uncertain due to reporting and will be subject to uncertainty due to the assumptions concerning the population immunity and changing in control efforts.

Response: We agree that there is more uncertainty in the beginning of an outbreak, when cases are more likely to have been missed, and thank the reviewer for bringing up this point. We acknowledge this limitation in lines 408-409: “…symptomatic cases may go unreported, particularly in the early stages of an outbreak, which could lead to an overestimate of the infectiousness of index cases.” We also agree that changes in population immunity and control efforts could also have contributed to the higher *R_Ei_* estimates in index cases. We acknowledge the former in lines 367-369: “…as an outbreak progresses and more individuals become ill and later immune, there is a natural decrease in the reproduction number” and the latter in lines 386-387: “rapid implementation of effective outbreak control measures could curtail transmission.”

Changes to text (line 369): Changed “proportion susceptible” to “reproduction number”.

Line 286: Any comment on why the influence of resident status should be affected by serial interval?

Response: It’s possible that staff and residents have different ‘true’ serial intervals. For example, if staff self-quarantine after their first symptoms by leaving the workplace, they could have a shorter infectious period and therefore shorter serial interval. Future studies with more detailed data on outbreak response should investigate this.

Line 387: Are there current guidelines for control of vomiting to compare these suggestions to?

Response: Yes, there are current guidelines for controlling nosocomial norovirus outbreaks. We thank the reviewer for raising this point. We have added reference 31 and added text to clarify the current recommendations.

Edits to text (lines 438-443): “While cleaning with a chlorine-based disinfectant and isolation of cases are currently recommended control strategies, there are generally little data about the implementation and timing of these control measures in norovirus outbreaks [10]. Furthermore, the use of antiemetic drugs for the prevention and control of norovirus outbreaks is not widely recommended [31, 32]. The important role of vomiting in transmission shown in our results suggests that the use of antiemetic drugs in nursing home outbreaks should be further considered.”

Edits to text (lines 558-560): “31. MacCannell T, Umscheid CA, Agarwal RK, Lee I, Kuntz G, Stevenson KB, et al. Guideline for the prevention and control of norovirus gastroenteritis outbreaks in healthcare settings. Infect Control Hosp Epidemiol. 2011;32(10):939-69. Epub 2011/09/21. doi: 10.1086/662025. PubMed PMID: 21931246.”

Reference 22: Appears incorrectly formatted

Response: We apologize for this formatting error. The reference has been updated.

Edits to text (lines 529-530): 22. Cori A. EpiEstim: a package to estimate time varying reproduction numbers from epidemic curves. R package version 1.1-2 ed2013.

Reviewer #3:

Major comments:

None

Minor comments:

Intro: the distinction between LTCFs and nursing homes may need to be clarified.

Response: We agree that this would be helpful to clarify and appreciate the suggestion. We added text to clarify the distinction.

Edits to text (lines 80-81): “…gastroenteritis outbreaks are common in nursing homes, skilled nursing facilities and assisted living facilities, which are collectively known as long-term care facilities (LTCFs) [4-7].”

Some limitations of the study merit mentioning:
• Absence of genotype information from some outbreaks (and thus the generalizability to all norovirus outbreaks)

Response: We agree that this is an additional limitation of the study and thank the reviewer for raising this point. We added text to specify that lack of genotype information is an additional limitation.

Edits to text (lines 405-407): “Additionally, because all outbreaks were caused by genogroup II, and the two outbreaks genotyped were caused by GII.4 Sydney, results may also not be generalizable to non-genogroup II or non-GII.4 outbreaks.”

• Absence of data on age and gender due to a large amount of missing data. This is problematic as some of the observed associations may be linked to age (and less probably gender). Can the authors not check this using only the cases with available data?

Response: We agree that it would be beneficial to examine age and/or sex as potential confounders using cases with available information and thank the reviewer for the suggestion. We examined these variables as potential confounders and found no evidence of confounding. We’ve added text to show this. Additionally, because we examined norovirus transmission in nursing homes, the age range for cases was generally quite narrow, so it’s unclear if age differences could meaningfully affect transmission.

Edits to text (lines 172-174): “However, a sensitivity analysis was performed using cases with available information on age and/or sex to examine whether these could be potential confounding variables.”

Edits to text (lines 315-326): “Furthermore, in sensitivity analyses including sex and/or age in the model, we found limited evidence for confounding of the association between vomiting and case infectiousness. When sex and age were included in the weighted mixed linear model, the association was slightly attenuated: cases who vomited infected 1.90 (95% CI: 1.36, 2.66) times the number of individuals as non-vomiters. However, conclusions about the relative importance of vomiting remained the same. Associations between case infectiousness and diarrhea and resident largely disappeared: cases with diarrhea infected 1.09 (95% CI: 0.77, 1.55) times the number of individuals as cases without diarrhea and resident-cases infected 0.67 (95% CI: 0.25, 1.76) times the number of individuals as staff-cases, suggesting possible confounding by sex and/or age. However, in univariate analyses between the outcome variable, exposure variables, and potential confounders, only the association between resident and age was statistically significant.”

Edits to text (lines 348-351): “While conclusions about the importance of vomiting in transmission remained the same when sex and age were included in the model, potential confounding of associations between case infectiousness and resident and diarrhea by sex and age should be further explored.”
