# Supplementary material for: Quantifying the roles of vomiting, diarrhea, and residents vs. staff in norovirus transmission in U.S. nursing home outbreaks
Source: PLoS Comput Biol. 2020 Mar 25;16(3):e1007271. doi: 10.1371/journal.pcbi.1007271 (PMC7135310; doi:10.1371/journal.pcbi.1007271)
Supplement: Attachment — Submitted filename: Second review 122819.docx [file pcbi.1007271.s008.docx]

I appreciate the thoughtful, informative and courteous reply of Adams et al. to all of the reviewer comments. The only lingering concerns I have are listed below. However, I would not classify any of these concerns as major.

- **“Line 253. Please clarify why the p-value is so small even when there is significant overlap of the IQR ranges. Response: Because we used the Kruskal-Wallis test, which is a non-parametric test that pools the data (across groups) and ranks observations from lowest to highest, it is possible to have statistically significant results even if IQRs overlap.”.** I am not a statistician (so I am happy to be corrected), but based on this description of the Kruskal-Wallis test it sounds like the test just identifies a two distributions as being statistically distinct, but not necessarily that they have a difference in a mean, median etc. However, it seems that the statistic is used in this analysis to show a statistically significant difference in mean/median. (For example, suppose you had two normal distributions centered around 0, but the first with a standard deviation of .1 and the second with a standard deviation of 10 – then it seems the p-value of the KW test would be really small, even though the true mans are the same?)

- **“Line 257. Did you try including an interaction term for vomiting and diarrhea … Response: Yes, we did try including an interaction term for vomiting and diarrhea. However, due to … problems with collinearity, we decided to exclude the interaction term from the model.”** Again, I am not a statistician, but my understanding is that you have to be careful about interpreting regression results when there are collinear terms, because the results can be very sensitive to minor alterations of data (i.e. similar data may reveal diarrhea is more important than vomiting). To circumvent this, I wonder if it would be better to dichotomize the regression with non-overlapping terms such as ‘vomiting-and-diarrhea’, ‘vomiting-only’, and ‘diarrhea-only’ rather than with ‘diarrhea’ and ‘vomiting’.

- Lines 157-59 (of revision). Thanks for clarifying the definition of C. Perhaps there are a couple typos though? Specifically, should the ‘1’ in the equation precede the fraction rather than be in the numerator? And should ‘where C is the total’ be ‘where C_1_ is the total’?

- Fig 2 (of revision). Thanks for making the changes. I just had trouble differentiating the colors (to my eye, it looks like there are just 3 colors and not 4).

- Lines 396-397 (of revision) “Conversely, more self-sufficient residents may be more likely to identify symptoms of norovirus and self-quarantine, thus transmitting less.” Thanks for including this point – I was actually thinking the mobility and self-sufficiency of the staff might lead to more staff self-quarantine and thus lower R for staff.
