# Supplementary material for: Quantifying the roles of vomiting, diarrhea, and residents vs. staff in norovirus transmission in U.S. nursing home outbreaks
Source: PLoS Comput Biol. 2020 Mar 25;16(3):e1007271. doi: 10.1371/journal.pcbi.1007271 (PMC7135310; doi:10.1371/journal.pcbi.1007271)
Supplement: Attachment — Submitted filename: Second Response to Reviewers.docx [file pcbi.1007271.s009.docx]

Norovirus Transmission in Nursing Homes manuscript

PLOS Computational Biology Revisions

Updated: January 15, 2020

**Requests from the reviewers:**

Reviewer #1:

- **“Line 253. Please clarify why the p-value is so small even when there is significant overlap of the IQR ranges. Response: Because we used the Kruskal-Wallis test, which is a non-parametric test that pools the data (across groups) and ranks observations from lowest to highest, it is possible to have statistically significant results even if IQRs overlap.”.** I am not a statistician (so I am happy to be corrected), but based on this description of the Kruskal-Wallis test it sounds like the test just identifies a two distributions as being statistically distinct, but not necessarily that they have a difference in a mean, median etc. However, it seems that the statistic is used in this analysis to show a statistically significant difference in mean/median. (For example, suppose you had two normal distributions centered around 0, but the first with a standard deviation of .1 and the second with a standard deviation of 10 – then it seems the p-value of the KW test would be really small, even though the true mans are the same?)

Response: Thank you for bringing this to our attention. We agree that we cannot, in fact, assume that that the R_Ei_ distributions of for all groups are identically shaped and scaled, and therefore cannot conclude that median R_Ei_ estimates are or are not significantly different using this method. Therefore, we removed the description of the Kruskal-Wallis test from the methods section and removed p-values from the results section that were calculated using the Kruskal-Wallis test. These include p-values for the following differences in median R_Ei_ estimates: vomit vs. no vomit, diarrhea vs. no diarrhea, both vomiting and diarrhea vs. diarrhea only, both vomiting and diarrhea vs. vomiting only, and residents vs. staff, and cases with R_Ei_ ≥ 1 vs. R_Ei_ < 1.

Edits to text (lines 269-270): “…however the interquartile ranges were largely overlapping.”

Edits to text (line 275): “Interquartile ranges were again overlapping for all comparisons.”

- **“Line 257. Did you try including an interaction term for vomiting and diarrhea … Response: Yes, we did try including an interaction term for vomiting and diarrhea. However, due to … problems with collinearity, we decided to exclude the interaction term from the model.”** Again, I am not a statistician, but my understanding is that you have to be careful about interpreting regression results when there are collinear terms, because the results can be very sensitive to minor alterations of data (i.e. similar data may reveal diarrhea is more important than vomiting). To circumvent this, I wonder if it would be better to dichotomize the regression with non-overlapping terms such as ‘vomiting-and-diarrhea’, ‘vomiting-only’, and ‘diarrhea-only’ rather than with ‘diarrhea’ and ‘vomiting’.

Response: Thank you for raising this issue. We agree that collinearity between predictor variables can lead to unreliable parameter estimates. However, after dropping the interaction term between vomit and diarrhea from our model, there were no longer issues of collinearity (i.e., vomiting and diarrhea were not collinear). Therefore, we believe our parameter estimates are reliable.

To examine a potential interaction between vomiting and diarrhea, we created dummy variables (as you suggest) for vomiting only, diarrhea only, both vomiting and diarrhea, and no vomiting or diarrhea, with no vomiting or diarrhea used as the reference group. Results were largely the same: cases with vomiting alone infected 2.09 (95% CI: 1.17, 3.71) times the number of individuals as cases with no vomiting or diarrhea, cases with diarrhea alone infected 1.37 (95% CI: 0.78, 2.41) times the number of individuals as cases with no vomiting or diarrhea, cases with both vomiting and diarrhea infected 2.90 (95% CI: 1.50, 5.62) times the number of individuals as cases with no vomiting or diarrhea, and resident-cases still infected 1.53 (95% CI: 1.15, 2.02) times the number of individuals as staff-cases. While this shows that there may haven been a slight interaction between vomiting and diarrhea, conclusions about the relative importance of vomiting compared to diarrhea remained the same. For this reason, and because only two individuals reported no vomiting or diarrhea, leading to much more uncertain results, we decided to keep the findings from our original regression analysis in the paper.

- Lines 157-59 (of revision). Thanks for clarifying the definition of C. Perhaps there are a couple typos though? Specifically, should the ‘1’ in the equation precede the fraction rather than be in the numerator? And should ‘where C is the total’ be ‘where C_1_ is the total’?

Response: Yes, thank you for pointing this out, we apologize for the error and have made the suggested changes to the equation.

Edits to text (line 157): $p_{i}= 1-\frac{\sum_{1}^{i} C_{i}}{C_{1}}$

Edits to text (line 158): “… where C_1_ is the total number susceptible on day 1…”

- Lines 396-397 (of revision) “Conversely, more self-sufficient residents may be more likely to identify symptoms of norovirus and self-quarantine, thus transmitting less.” Thanks for including this point – I was actually thinking the mobility and self-sufficiency of the staff might lead to more staff self-quarantine and thus lower R for staff.

Response: Thank you for clarifying. We agree that quick, self-quarantining of staff (i.e., recognizing symptoms and staying home from work) would lead to lower R_Ei_ for staff, however the level of mobility and self-sufficiency for staff, unlike residents, is likely fairly homogenous, as staff must be mobile and self-sufficient to be able to work at the nursing home.
